# The WNT/β-catenin pathway regulates expression of the genes involved in cell cycle progression and mitochondrial oxidative phosphorylation in the postmitotic cardiac myocytes

**DOI:** 10.20517/jca.2021.35

**Published:** 2022-01-28

**Authors:** Melis Olcum, Sirisha M. Cheedipudi, Leila Rouhi, Siyang Fan, Hyun-Hwan Jeong, Zhongming Zhao, Priyatansh Gurha, Ali J. Marian

**Affiliations:** 1 Center for Cardiovascular Genetics, Institute of Molecular Medicine and Department of Medicine, University of Texas Health Sciences Center at Houston, Houston, TX 77030, USA.; 2 Heart Center & Beijing Key Laboratory of Hypertension, Beijing Chaoyang Hospital, Capital Medical University, Beijing 100020, China.; 3 Center for Precision Health, School of Biomedical Informatics and School of Public Health, The University of Texas Health Science Center at Houston, Houston, TX 77030, USA.

**Keywords:** Myocyte proliferation, WNT signaling, beta-catenin, cytokinesis, cell cycle, oxidative phosphorylation, mitochondria

## Abstract

**Introduction:**

Aging is associated with cardiac myocyte loss, sarcopenia, and cardiac dysfunction. Adult cardiac myocytes are postmitotic cells with an insufficient proliferative capacity to compensate for myocyte loss. The canonical WNT (cWNT) pathway is involved in the regulation of cell cycle reentry in various cell types. The effects of the cWNT pathway on the expression of genes involved in cell cycle reentry in the postmitotic cardiac myocytes are unknown.

**Aim:**

The aim of the study was to identify genes whose expression is regulated by the β-catenin, the indispensable component to the cWNT signaling, in the postmitotic myocytes.

**Methods and Results:**

Cardiac myocyte-specific tamoxifen-inducible MerCreMer (*Myh6-Mcm*) mice were used to delete the floxed exon 3 or exons 8 to 13 of the *Ctnnb1* gene to induce gain-of-function (GoF) or loss-of-function (LoF) the β-catenin, respectively. Deletion of exon 3 leads to the expression of a stable β-catenin. In contrast, deletion of exons 8–13 leads to the expression of transcriptionally inactive truncated β-catenin, which is typically degraded. GoF or LoF of the β-catenin was verified by reverse transcription-polymerase chain reaction (RT-PCR), immunoblotting, and immunofluorescence. Myocyte transcripts were analyzed by RNA-Sequencing (RNA-Seq) at 4 weeks of age. The GoF of β-catenin was associated with differential expression of ~1700 genes, whereas its LoF altered expression of ~400 genes. The differentially expressed genes in the GoF myocytes were enriched in pathways regulating the cell cycle, including karyokinesis and cytokinesis, whereas the LoF was associated with increased expression of genes involved in mitochondrial oxidative phosphorylation. These findings were validated by RT-PCR in independent samples. Short-term GoF nor LoF of β-catenin did not affect the number of cardiac myocytes, cardiac function, myocardial fibrosis, myocardial apoptosis, or adipogenesis at 4 weeks of age.

**Conclusion:**

Activation of the β-catenin of the cWNT pathway in postmitotic myocytes leads to cell cycle reentry and expression of genes involved in cytokinesis without leading to an increase in the number of myocytes. In contrast, suppression of the β-catenin modestly increases the expression of genes involved in oxidative phosphorylation. The findings provide insights into the role of β-catenin of the cWNT pathway in the regulation of cell cycle reentry and oxidative phosphorylation in the postmitotic cardiac myocytes.

## INTRODUCTION

Aging is associated with a drop in the number of cardiac myocytes, sarcopenia, and mitochondrial dysfunction among others, collectively, leading to impaired cardiac function (reviewed in Ref.^[1]^). Likewise, myocyte loss is the core component of various myocardial pathological conditions, including myocardial infarction and heart failure, which are major causes of mortality and morbidity in the elderly population. To compensate for the loss, the cardiac myocytes need to replicate. However, cardiac myocytes progressively lose their proliferative capacity and become postmitotic with a renewal rate of < 1% per year^[2]^. Consequently, there is considerable interest in forcing the postmitotic myocytes to re-enter the cell cycle and proliferate.

The WNT/β-catenin pathway, known as the canonical WNT (cWNT) pathway, is an ancient evolutionarily conserved pathway involved in numerous biological processes, including embryogenesis, stem cell fate, cell proliferation, differentiation, cancer, and senescence^[3]^. The WNT pathway, discovered about 40 years ago, is activated upon binding of one of the 19 known WNT ligands to its cognate transmembrane co-receptors frizzled and lipoprotein receptor-related protein 5/6 (LRP5/6). The WNT molecules are secreted glycoproteins, whose secretion requires palmitoylation by the porcupine enzyme. Inhibition of porcupine effectively suppresses the WNT pathway and has been used as a potential therapy in cardiomyopathies^[4]^. The binding of the WNT molecule to its co-receptors induces the formation of the WNT signaling complex. The complex protects the β-catenin from degradation by the destruction complex, enabling its nuclear localization. In the nucleus, the β-catenin binds to the T-cell factor/lymphoid enhancer-binding factor (TCF/LEF) transcription factors and serves as the co-transcriptional regulators of the expression of the cWNT target genes. In the absence of the WNT ligands, the destruction complex, comprised of axis inhibition (AXIN), glycogen synthase kinase 3β (GSK3β), casein kinase 1α(CK1α), adenomatous polyposis coli (APC), target the β-catenin for sequential phosphorylation, initially by CK1α at Ser 45 and subsequently by GSK3β at Thr41, Ser37, and Ser 33 at the N terminal domain. The phosphorylated β-catenin is recognized by the β-transducin repeats-containing protein/Skp-Cullin1-F-Box complex and is subjected to degradation by the ubiquitin-proteasome system (UPS).

In the heart, the cWNT pathway is best known to regulate cardiac development and proliferation of myocytes during the embryonic period^[5]^. The pathway also regulates the responses of the heart to injury, including myocardial fibrosis and cardiac remodeling post-myocardial infarction and pressure overload-induced cardiac hypertrophy^[6,7]^. Furthermore, the cWNT pathway is implicated in the pathogenesis of fibro-adipogenesis in arrhythmogenic right ventricular cardiomyopathy, a hereditary disease caused by mutations in genes encoding desmosome proteins^[4,8–10]^. Desmosomes are members of the intercalated discs and are responsible for myocardial mechanical integrity, mechano-sensing, and mechano-transduction, including regulation of the Hippo and the cWNT pathways^[11–13]^. The β-catenin is a major constituent of the intercalated disc proteins and has functions independent of the cWNT pathway^[14]^.

Several studies have targeted the cWNT pathway for beneficial gains in various cardiovascular pathological states, including cardiomyopathies (reviewed in Ref.^[15]^). Despite the intense interest in the role of the cWNT pathway in myocyte proliferation, differentiation, maturation, aging, and various cardiovascular pathology, the transcriptional signature of this major pathway in mature cardiac myocytes has remained unknown. The purpose of the present study was to identify genes whose expressions are regulated by the cWNT/β-catenin pathway in postnatal quiescent cardiac myocytes using the genetic gain-of-function (GoF) and loss-of-function (LoF) approaches followed by sequencing of the cardiac myocyte transcripts.

## METHODS

### Regulatory approval

The animal experiments were conducted according to the NIH Guide for the Care and Use of Laboratory Animals and were approved by the The University of Texas Health Science Center-Houston: AWC-21-0015.

### Genetic suppression or activation of the β-catenin specifically in cardiac myocytes

The cWNT/β-catenin pathway was activated or suppressed using previously characterized genetic models of inducible GoF and LoF of β-catenin (CTNNB1), as β-catenin is the key indispensable nuclear effector of the cWNT pathway [Supplementary Figure 1]^[3,16–19]^. Deletion of exon 3 of the *Ctnnb1* gene by removing the GSK3β phosphorylation sites (Thr41, Ser37, and Ser33), stabilizes the β-catenin protein, and activates the cWNT/β-catenin pathway (GoF)^[20]^. In contrast, deletion of the transactivation motif (amino acids 361–692), encoded by exons 8 to 13 in the *Ctnnb1* gene, eliminates transcriptional activity of the β-catenin, typically resulting in the degradation of the truncated protein and leading to LoF of the cWNT/β-catenin pathway [Supplementary Figure 1]. Fidelity of the GoF [*Ctnnb1*^F(ex3)^] (a gift from Dr. Takedo) and LoF [B6(Cg)-*Ctnnb1*^*tm1Knw*^/J, *Ctnnb1*^*F* (Ex8–13)^] mouse models has been established^[20–22]^. To suppress or activate the cWNT/β-catenin in cardiac myocytes, *Myh6-Mcm* and *Ctnnb1*^F/F(ex8–13^) or *Myh6-Mcm* and *Ctnnb1*^F/F(ex3^) mice were crossed, and the offspring carrying the desired genotypes, designated as *Myh6-Mcm:Ctnnb1*^LoF^ and *Myh6-Mcm:Ctnnb1*^GoF^, respectively, were treated with subcutaneous injection of tamoxifen (30 mg/kg/day) for 5 consecutive days starting from postnatal day 14 to induce deletion of the *Ctnnb1* gene specifically in cardiac myocytes [Supplementary Figure 1]^[17,21]^. The P14 time point was selected to avoid potential untoward effects on developing myocytes as, by P14, most of the cardiac myocytes lose their proliferative capacity and are considered quiescent.

### Genotyping

Genotyping was performed by PCR using genomic DNA isolated from the mouse tails, as described^[10,23]^. The list of oligonucleotide primers used for the genotyping is provided in Supplementary Table 1.

### Isolation of cardiac myocytes

Cardiac myocytes were isolated from 4-week-old mice in the experimental groups as published^[23–25]^. In brief, mice were anesthetized using pentobarbital, the heart was excised, cannulated retrogradely, and perfused at a flow rate of 4 mL/min with a digestion buffer containing type 2 collagenase at 2.4 mg/mL concentration (Worthington Cat# LS004176). Upon completion of the enzymatic digestion, the ventricles were freed from the large blood vessels and minced in a stop buffer containing 10% calf serum, 12.5 μM CaCl_2_, and 2 mM ATP. Then the cell suspension was filtered through a 100 μm cell strainer and centrifuged at 20 G to precipitate the cardiac myocytes. This was followed by the re-introduction of calcium by a stepwise increase in the molar concentration of CaCL_2_ from 100 μM to 400 μM and to 900 μM in the stop buffer. Upon re-introduction of the last CaCl_2_ concentration, cardiac myocytes were suspended either in a Qiazol reagent (Qiagen, Cat# 79306) for RNA extraction or in a protein extraction buffer for immunoblotting.

### RNA-Sequencing

To identify genes that are differentially expressed upon suppression or activation of the β-catenin, cardiac myocyte transcripts were analyzed by RNA-Seq, as published^[23,26]^. In brief, total RNA was extracted from mouse ventricular cardiac myocytes using the miRNeasy Mini Kit (Qiagen, Cat# 217004). RNA extracts with an RNA Integrity Number value of > 8, determined using an Agilent Bioanalyzer RNA chip, were used to construct sequencing libraries. Strand-specific ribosomal RNA depleted sequencing libraries were generated using TruSeq stranded total RNA library preparation kit (Illumina Inc., Cat# 20020596). The libraries were sequenced as 75bp paired-end reads on an Illumina HiSeq 4000 instrument.

### Bioinformatics analysis of the RNA-Seq data

The quality of RNA sequence reads was assessed by FASTQC, and the STAR program was used for alignment of the sequence reads to the mouse reference genome build mm10 and generation of gene read counts^[27]^. The GENCODE gene model was used to annotate the uniquely aligned read pairs (https://www.gencodegenes.org/mouse/). The differentially expressed genes (DEGs) were identified using the DESeq2 statistical package in R^[28]^. Benjamini-Hochberg FDR-adjusted *P*-value of < 0.05 were considered significant. The R packages were used for the visualization of heatmaps and volcano plots (www.rstudio.com). The GOCHORD function in R was used to plot the Circos maps.

Gene Set Enrichment (GSEA, version 2.2.3, http://software.broadinstitute.org/gsea/) was used to predict dysregulated biological pathways. Normalized Enrichment Score determined from the Molecular signature database (MSigDB) 3.0 was used to curate gene sets for the Hallmark canonical pathways. The DEGs were analyzed using the upstream regulator analysis function of the Ingenuity Pathway Analysis software (IPA^®^, QIAGEN Redwood City) to predict their transcriptional regulators (TRs). IPA was used to identify the targets of the specific TRs. TRs showing a *P*-value of < 0.05 for overlap with the IPA target genes and a predicted Z score of < −2 or > 2 were considered dysregulated.

### Survival analysis

Kaplan Meier survival rates were constructed for the wild type (WT), *Myh6-Mcm*, *Myh6-Mcm*:*Ctnnb1*^LoF^, *Myh6-Mcm*:*Ctnnb1*^GoF^ mice and plotted using GraphPad Prism 8 software (https://www.graphpad.com/).

### Gross morphology

Body and heart weight were measured at 4 weeks of age, and the ratios of the heart weight indexed to bodyweight were compared among the groups.

### Echocardiography

Echocardiography was performed at 4 weeks of age using a Vevo 1100 ultrasound imaging system (FUJIFILM VisualSonics Inc., Toronto, ON, Canada), as published^[23,26]^. In brief, B-mode guided M mode images of the ventricles were obtained in mice lightly anesthetized with 1% isoflurane. Anterior wall thickness, posterior wall thickness (PWT), left ventricular end-systolic diameter (LVESD), and left ventricular end-diastolic diameter (LVEDD) were measured using the leading-edge method. The measurements were used to calculate the left ventricular fractional shortening and left ventricular mass. The latter was calculated using the Devereux formula, as LVM=0.8 × [1.04 × (ST + LVEDD + PWT)^3^ − (LVEDD)^3^] and indexed to body weight^[29]^.

### Electrocardiography

Two-lead surface electrocardiogram was recorded for about an hour while keeping the mice under light anesthesia, as published^[24,25]^. The rhythm was recorded using a Power Lab 4/30 data acquisition system, and the data were analyzed using Lab Chart7 software (ADInstruments, Colorado Springs, CO, USA).

### Myocardial fibrosis

To detect and quantify myocardial fibrosis, thin myocardial sections were stained with picrosirius red, and the percent area stained, as a measure of collagen volume fraction (CVF), was calculated as published^[23,24]^. CVF was calculated using the Image J software in 8 high magnification microscopic fields (×40) per section, in 6 sections per heart, and a minimum of 5 mice per group.

### Myocardial adipocytes

To detect adipocytes in the heart, thin myocardial sections were incubated in 4% formaldehyde solution, subjected to antigen retrieval in a sodium citrate buffer (pH 6.0), and incubated with an antibody against Perilipin 1 (PLIN1), a marker of lipid-containing adipocytes (Cell Signaling Technology, Cat# 9349), as published^[24]^. Following overnight incubation, sections were treated with the secondary antibody conjugated to Alexa 594, and the nuclei were counterstained with 4’, 6 Diamidino-2-phenylindole dihydrochloride (DAPI, Sigma-Aldrich St Louis, MO; Cat# D8417) at 1 μg/mL concentration. The stained sections were mounted using fluorescence mounting media (DAKO, Cat# S3023) and imaged using a Zeiss Axioplan fluorescence microscope. The total number of PLIN1-stained adipocytes were counted in each section, in 6 sections per mouse, and at least 5 mice per genotype. The average number of cells stained positive for PLIN1 expression per myocardial section was determined per group and compared among the genotypes.

### Myocyte cross-sectional area

To quantitate myocyte cross-sectional area (CSA), thin myocardial sections were stained with wheat germ agglutinin (WGA) to mark the interstitium, and an antibody against pericentriolar material protein 1 (PCM1), which marks the myocyte nuclei (Sigma, Cat# HPA023370), as published^[23,24,30,31]^. In brief, thin myocardial sections (5 μm) were incubated with WGA (1 μg/mL) conjugated to Texas red (Thermo Fisher Scientific, Cat# W21405) to mark the interstitium. Sections were co-stained with an anti PCM1 antibody and DAPI. The WGA and PCM1 co-stained images were analyzed by Image J software (https://imagej.net) to measure the interstitial area and to count the number of cardiac myocytes in each field, normalized to the nuclei stained positive for PCM1 expression. An average of 8 fields per section, 6 sections per heart, representing around 20,000 nuclei per heart was analyzed.

### Myocardial apoptosis

To detect apoptosis, the terminal deoxynucleotidyl transferase deoxyuridine triphosphate (dUTP) nick end labeling (TUNEL) assay was performed using In-Situ cell death detection Fluorescein kit (Roche, Cat# 11684795910), as published^[23,25]^. The nuclei stained positive for the TUNEL assay were identified in 8 high magnification fields (×40) per section, in 6 sections per heart, and 5 mice per group. About 20,000 nuclei per mouse heart were counted to determine the percentage of nuclei stained positive for TUNEL, and the mean values were compared among the groups.

### Immunoblotting

Immunoblotting was performed on cardiac myocytes and whole heart protein extracts, as published^[9,26,32]^. In brief, to extract proteins, ventricular tissues or cardiac myocytes were homogenized and dissolved in a radioimmunoprecipitation assay buffer (Cat# 974821) containing 0.5% SDS along with protease and phosphatase inhibitors (Roche, Cat# 04693159001, Cat# 04906837001, respectively). The lysates were sonicated briefly using a Bioruptor Pico (Diagenode) and centrifuged at 13,000 rpm to precipitate the protein. Aliquots of 50 μg total protein were loaded onto a 10% SDS polyacrylamide gel, electrophoresed, and transferred onto a nitrocellulose membrane. The membranes were probed with antibodies against the target proteins. The corresponding secondary antibodies were used, and the signals were detected using the ECL western blotting detection kit (Amersham, Cat# RPN2106) and were imaged using the LiCOR (Odyssey) imaging system. All antibodies used are listed in Supplementary Table 1.

### Reverse transcription-polymerase chain reaction

To quantitate transcript levels of selected genes, total RNA was extracted from myocytes using a miRNeasy Mini Kit (Qiagen, Cat# 217006) and treated with DNAse I (Qiagen, Cat# 79254) as per the manufacturer’s instructions. Reverse transcription was performed with random primers in approximately 1 μg of total RNA using a high-capacity cDNA synthesis kit (Applied Biosystems, Cat# 4368814). Transcript levels of the selected genes were determined using SYBR green or TaqMan assays in duplicate reactions and normalized to the *Gapdh* or *Vcl* transcript levels. Changes in the transcript levels were calculated using the ΔΔCT method and presented as fold changes relative to the values in the WT mice. The reverse transcription-polymerase chain reaction (RT-PCR) reactions were performed in 5 mice per group. The primers used in the study are listed in the Supplementary Table 1.

### Conventional statistical methods

The data were analyzed by the Shapiro-Wilk normality test, and the normally distributed data were compared between two groups using the *t*-test and among multiple groups by one-way ANOVA followed by Bonferroni pairwise comparisons. Data that departed from the Gaussian distribution were analyzed by the Kruskal-Wallis test. The categorical data were analyzed by the Fisher exact or the Chi-Square test. Kaplan-Meier survival plots were constructed and compared using the Log-rank test. Statistical analyses were performed using Graph pad Prism 8 or STAT IC, 15.1.

## RESULTS

### Genetic activation and suppression of the cWNT/β-catenin in the postnatal cardiac myocytes

**Figure 1.**
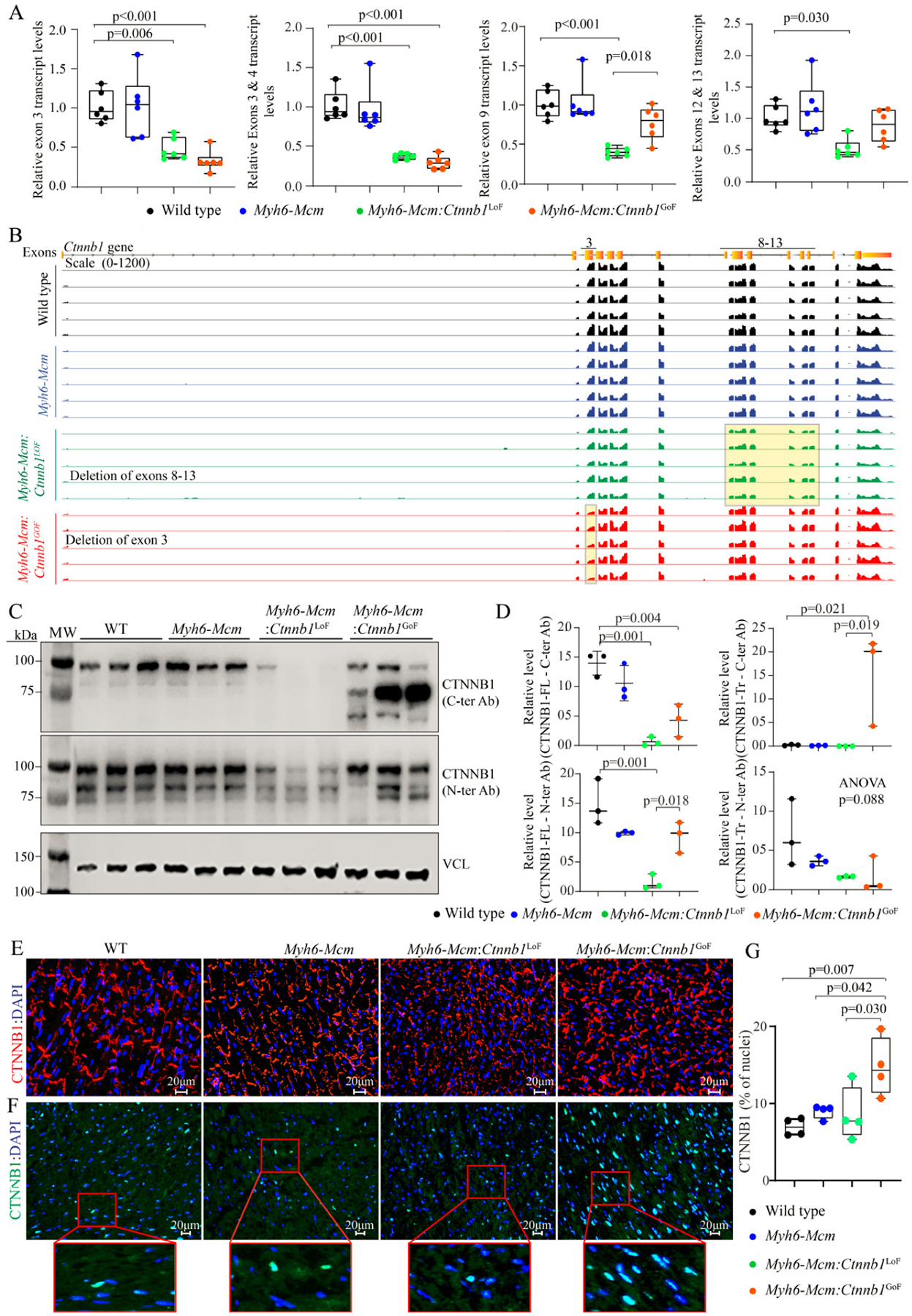
Activation or suppression of the cWNT/β-catenin pathway in the postmitotic cardiac myocytes. (A) RT-PCR data showing the transcript levels of *Ctnnb1* in the β-catenin gain-of-function (GoF) and loss-of-function (LoF) cardiac myocytes compared to wild type and *Myh6-Mcm* myocytes using primers designed to specifically amplify the exon 3, exon 3–4, exon 9, and exon 12–13 of the *Ctnnb1* gene. (B) IGV Genome browser images of read counts of *Ctnnb1* transcript levels obtained from the RNA-Seq data of wild type, *Myh6-Mcm*, β-catenin GoF, and LoF cardiac myocytes. (C) Western blot showing expression of the CTNNB1 protein in the cardiac myocyte protein extracts from wild type (WT), *Myh6-Mcm*, *Myh6-Mcm:Ctnnb1*^LoF^, and *Myh6-Mcm:Ctnnb1*^GoF^ mice, detected using antibodies raised against N- or C-terminal region of the β-catenin protein. The lower panel represents an immunoblot showing the expression of vinculin protein, which was used as the corresponding loading control. (D) Graphs depicting quantitative data of the CTNNB1 protein levels in the wild type, *Myh6-Mcm*, *Myh6-Mcm:Ctnnb1*^LoF^, and *Myh6-Mcm:Ctnnb1*^GoF^ cardiac myocyte protein extracts normalized to the vinculin protein levels (*n* = 3). (E) Representative immunofluorescence panels showing expression and localization of the CTNNB1 protein to the intercalated disks in the experimental groups. (F) Nuclear localization of CTNNB1 as detected by staining of thin myocardial sections with an anti CTNNB1 antibody. The number of nuclear stained positive for the CTNNB1 as well as the intensity of the fluorescence signaling were increased in the GoF mouse hearts, whereas the number of nuclei expressing CTNNB1 was not significantly changed in the LoF mouse hearts, albeit the signal intensity of the CTNNB1 was weaker. (G) Quantitative data showing the number of nuclei staining positive for the expression of CTNNB1. RT-PCR: Reverse transcription-polymerase chain reaction; *Myh6-Mcm*: myosin heavy chain 6-MerCreMer; RNA-Seq: RNA-Sequencing.

The β-catenin was genetically activated or suppressed specifically in the postnatal cardiac myocytes upon daily administration of tamoxifen to *Myh6-Mcm*:*Ctnnb1*^LoF^ and *Myh6-Mcm*:*Ctnnb1*^GoF^ mice from P14 to P19 [Supplementary Figure 1]. Postnatal targeting of the β-catenin at P14 was selected to avoid the known effects of the cWNT/β-catenin pathway on myocyte proliferation during early postnatal periods. The efficiency of suppression or activation of the β-catenin was analyzed by RT-PCR, mapping of the transcript reads in the RNA-Seq data, immunoblotting, and immunofluorescence studies. Cre-mediated excision of the specific exons was analyzed by quantifying transcript levels of the specific floxed exons by RT-PCR using 2 sets of primers for each genotype as well as in the RNA-Seq data. In accord with the Cre-mediated excision of the exons, transcripts containing exon 3 and exons 8–13 were markedly reduced in the hearts isolated from β-catenin GoF and LoF mouse models, respectively [Figure 1A]. In addition, the transcripts levels of the non-floxed exons of the *Ctnnb1* gene were also reduced in the *Myh6-Mcm*:*Ctnnb1*^LoF^ myocytes, likely reflective of the nonsense-mediated decay of the truncated transcripts [Figure 1A]. In contrast, transcript levels of the non-floxed exons of the *Ctnnb1* gene were unchanged in the *Myh6*-*Mcm*:*Ctnnb1*^GoF^ myocytes, indicating that the loss of exon 3 did not affect the *Ctnnb1* transcript stability [Figure 1A].

In accord with the RT-PCR data, the number of reads in the RNA-Seq data that was mapped to exon 3 of the *Ctnnb1* gene in the genome browser was significantly reduced in the *Myh6*-*Mcm*:*Ctnnb1*^GoF^ group [Figure 1B]. Likewise, the transcript levels of exons 8 to 13 of the *Ctnnb1* gene, which was floxed, as well as the non-floxed exons were reduced in the *Myh6-Mcm*:*Ctnnb1*^LoF^ myocytes, i.e., the read for all *Ctnnb1* exons were lower, consistent with the nonsense-mediated decay of the truncated transcripts [Figure 1B].

Levels of the β-catenin (CTNNB1) protein were increased markedly in the *Myh6-Mcm*:*Ctnnb1*^GoF^ mouse hearts, consistent with activation of the cWNT pathway [Figure 1C and D]. The size of the β-catenin protein was smaller than the size of full-size protein because of deletion of floxed exon 3, which removes about 80 amino acids (~10 kDa) in the *Myh6-MCM*:*Ctnnb1*^GoF^ mouse hearts. The level of the smaller β-catenin protein was elevated as the deletion removes the phosphorylation sites of GSK3β, which targets the protein for degradation by the UPS. A less intense band representing the full-length β-catenin was also detected, which reflects an incomplete Cre-mediated excision in the *Myh6-Mcm*:*Ctnnb1*^GoF^ mouse heart [Figure 1C]. In contrast, the truncated β-catenin (~60 kDa), resulting from the Cre-mediated excision of the exons 8–13 of the *Ctnnb1* gene corresponding to removal of about 270 amino acids, was not detected in the *Myh6-Mcm*: *Ctnnb1*^LoF^ mouse hearts, despite probing with two different antibodies [Figure 1C and D]. The finding likely reflects the quality control surveillance at the mRNA and protein levels, rendering the truncated mRNA and/or protein unstable. Expression of the full-length β-catenin protein was detected at low levels in the *Myh6-Mcm*:*Ctnnb1*^LoF^ mouse hearts, likely because of incomplete Cre-mediated excision of the floxed exons 8 to 13 of the *Ctnnb1* gene [Figure 1C and D].

Immunofluorescence staining of thin myocardial sections showed reduced localization of β-catenin to IDs in the *Myh6-Mcm*:*Ctnnb1*^LoF^ and increased density and size of the β-catenin-stained areas in the myocardium in the *Myh6-Mcm*:*Ctnnb1*^GoF^ mouse myocardial sections [Figure 1E]. To assess nuclear localization of the β-catenin, thin myocardial sections were stained with anti-β-catenin antibody that stains the nuclear but not junctional β-catenin. The number of DAPI-stained nuclei expressing the β-catenin protein as well as the intensity of the signal were increased in the *Myh6-Mcm*:*Ctnnb1*^GoF^ mouse hearts [Figure 1F and G]. Whereas there was no significant reduction in the number of nuclei expressing the β-catenin protein in the LoF genotype, the intensity of the β-catenin nuclear signal was weaker [Figure 1F and G].

### RNA-Seq quality control metrics

To identify genes whose expression levels were altered upon postnatal activation or suppression of the cWNT pathway specifically in cardiac myocytes, ribosome-depleted RNA was isolated from cardiac myocytes and sequenced. Administration of tamoxifen and activation of Cre transiently affect transcript levels of ~350 genes^[33]^. Therefore, to identify the transcriptomic signature of the cWNT/β-catenin pathway in cardiac myocytes, transcripts that are known to be affected by the tamoxifen-Cre system were removed before the analysis of the RNA-Seq dataset.

**Figure 2.**
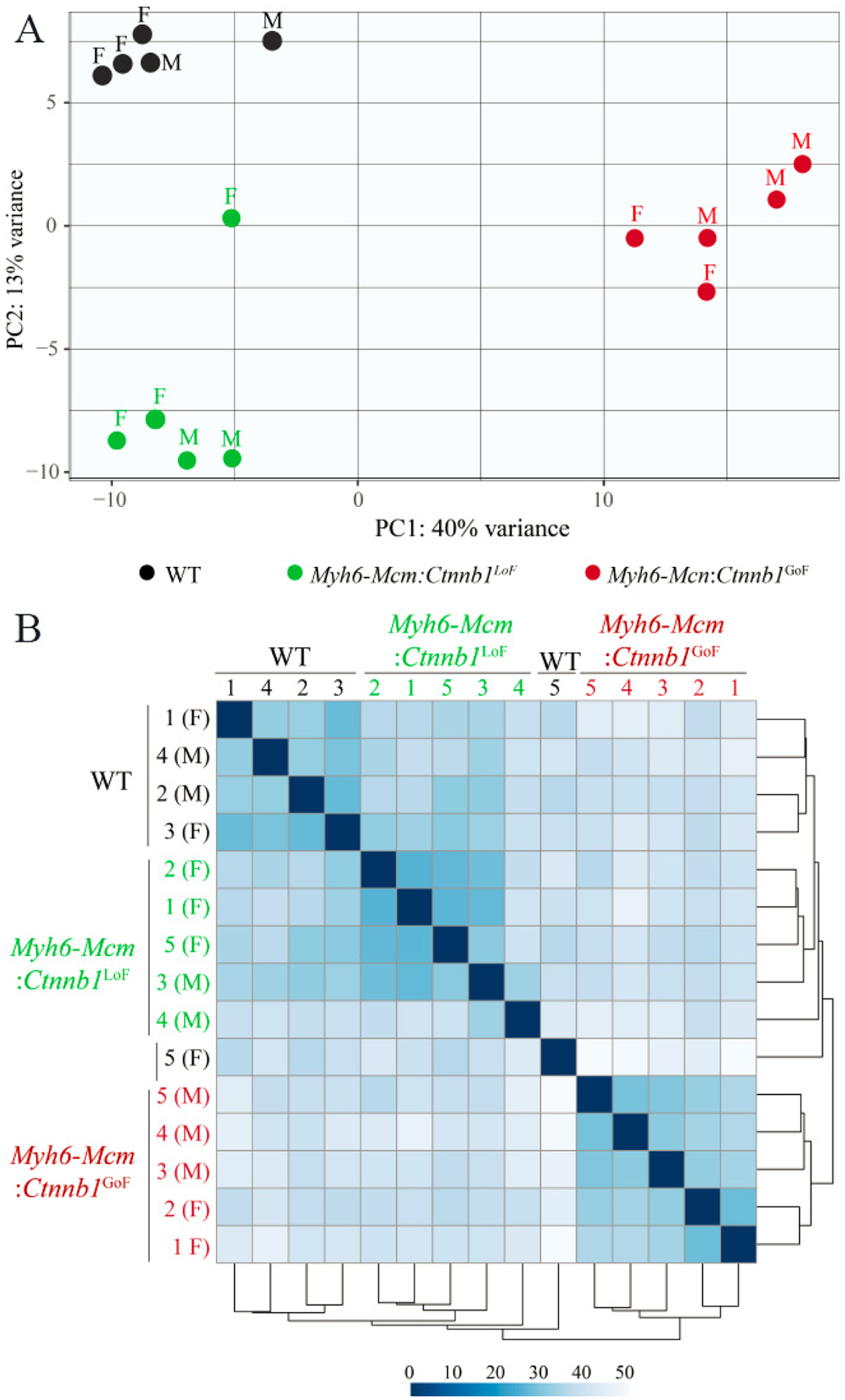
RNA-sequencing data depicting genotype-dependent clustering of cWNT/β-catenin activation or suppression in the cardiac myocytes. (A) Principal component analysis of cardiac myocyte transcriptome obtained through RNA-Seq data of wild type, β-catenin GoF and LoF showing distinct genotype-dependent segregation. (B) Correlation map of cardiac myocyte transcriptome in wild type, *Myh6-Mcm:Ctnnb1*^LoF^ and *Myh6-Mcm:Ctnnb1*^GoF^ hearts depicting strong correlation among the samples. *Myh6-Mcm*: Myosin heavy chain 6-MerCreMer; RNA-Seq: RNA-Sequencing; GoF: gain-of-function; LoF: loss-of-function.

The average number of total reads (54.3 ± 4.9 million reads), uniquely mapped reads (41.6 ± 4.1 million reads), and percent uniquely mapped reads (76.0% ± 2.3%) indicated a high quality of RNA-sequencing and did not differ among the genotypes [Supplementary Table 2]. Genotype-dependent clustering of the transcripts was assessed by Principal Component Analysis, which showed distinct separation of the myocyte transcripts among the three groups with the *Myh6-Mcm:Ctnnb1*^GoF^ forming the most distinct cluster [Figure 2A]. Likewise, there were strong correlations in the transcript levels among the samples within each genotype [Figure 2B].

### Effects of postnatal suppression or activation of the β-catenin on cardiac myocyte transcripts

**Figure 3.**
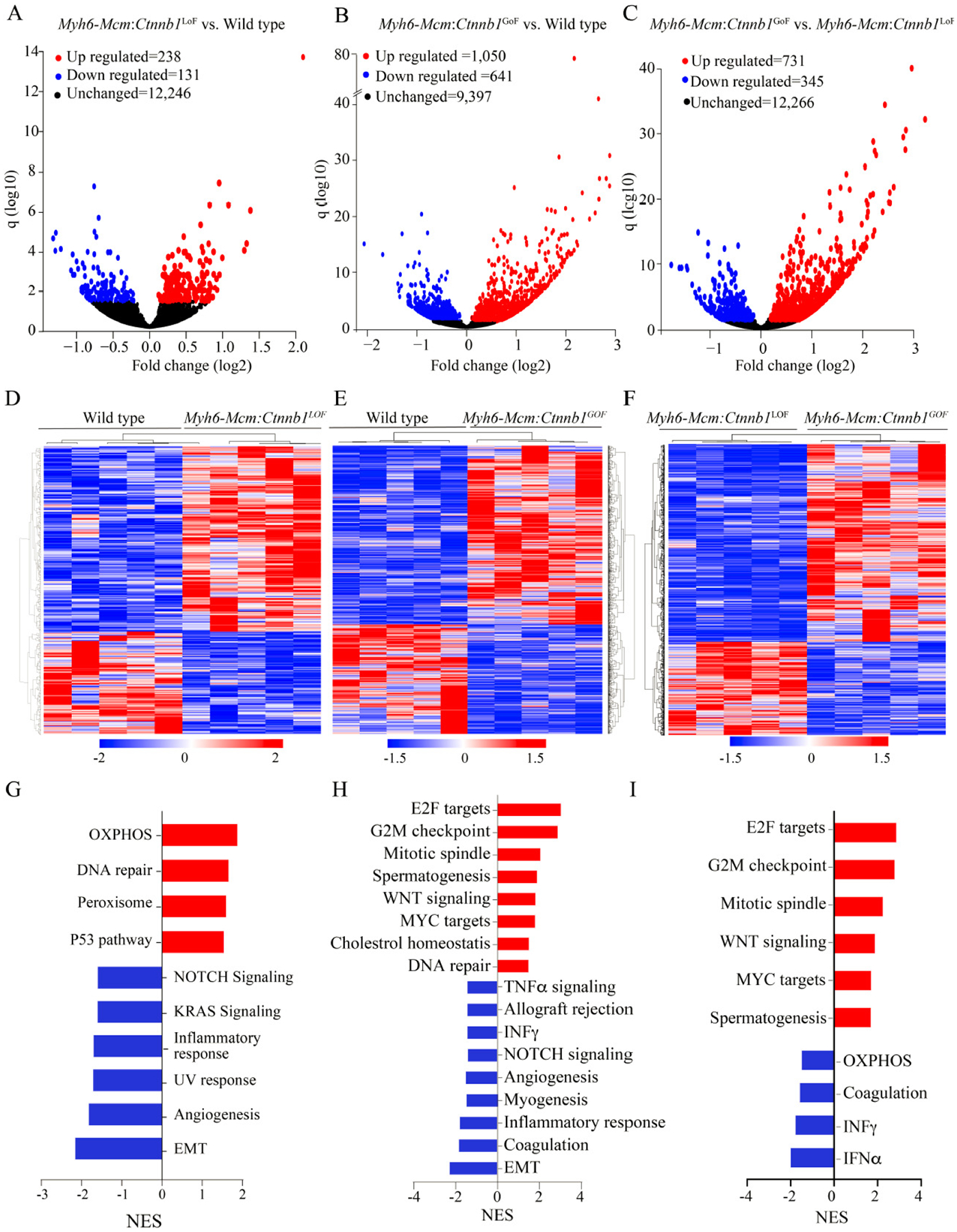
Differentially expressed genes (DEGs) in cardiac myocytes upon cWNT/β-catenin activation or suppression. (A) Volcano plot of DEGs showing 238 upregulated (red), 131 downregulated (blue) and 12,246 unchanged (black) genes upon suppression of the β-catenin in the cardiac myocytes compared to the wild type myocytes. (B) Volcano plot of the DEGs in the *Myh6-Mcm:Ctnnb1*^GoF^ cardiac myocytes compared to the wild-type myocytes. The upregulated DEGs are shown in red (1050), the downregulated in blue (641), and the unchanged genes in black (9397). (C) Volcano plot of the DEGs comparing the transcripts in the *Myh6-Mcm:Ctnnb1*^GoF^ and *Myh6-Mcm:Ctnnb1*^LoF^ myocytes showing 731 upregulated genes in red, 345 downregulated genes in blue, and 12,266 unchanged genes in black in the postmitotic cardiac myocytes. (D-F) Heat map of the DEGs between wild type *vs. Myh6-Mcm:Ctnnb1*^LoF^ (D), between wild type *vs. Myh6-Mcm:Ctnnb1*^GoF^ (E), and between *Myh6-Mcm:Ctnnb1*^GoF^ *vs. Myh6-Mcm:Ctnnb1*^LoF^ (F), which show distinct genotype-dependent clustering. (G) GSEA analysis of the DEGs in the *Myh6-Mcm:Ctnnb1*^LoF^ cardiac myocytes showing upregulated genes enriched in the oxidative phosphorylation (OXPHOS) pathway and downregulation of genes involved in the EMT pathway. (H) GSEA analysis of the DEGs depicting upregulated genes as being targets of E2F and mapped to the G2/M cell cycle checkpoint in the β-catenin activated cardiac myocytes. (I) GSEA analysis of the DEGs comparing the β-catenin activated and suppressed cardiac myocytes showing upregulated genes are enriched for the E2F targets and those involved in the G2/M checkpoint.

LoF of the *Ctnnb1* gene was associated with differential expression of 369 genes (238 upregulated and 131 downregulated), whereas GoF of the *Ctnnb1* gene led to dysregulation of ~1700 genes (1050 upregulated and 641 downregulated), as compared to the WT myocytes [Figure 3A and B]. Comparing the transcript levels between the myocytes from the GoF and LoF mice identified differential expression of about 1100 genes, comprising 731 upregulated and 345 downregulated genes [Figure 3C]. Heat maps corresponding to the pairwise comparison of the transcripts, depicting the DEGs are shown in Figure 3D–F. Gene set enrichment analysis predicted enrichment of genes involved in oxidative phosphorylation (OXPHOS) in the LoF myocyte transcripts, whereas the E2F target genes and genes involved in G2M checkpoints were enriched in the GoF myocyte transcripts [Figure 3G–H]. Comparing the LoF and GoF myocyte transcripts mainly illustrated changes similar to the comparison of the GoF and WT myocytes [Figure 3I]. Likewise, GSEA for KEGG pathways identified a similar set of biological pathways dysregulated, namely OXPHOS and cell cycle pathways, in the myocytes isolated from mice with the LoF and GoF of the *Ctnnb1* gene, respectively [Supplementary Figure 2]. IPA of the DEGs predicted SOX2 as the most activated and KDM5A, as the most suppressed transcriptional regulators of gene expression in the LoF myocytes [Supplementary Figure 3A]. In the GoF myocytes, the DEGs predicted activation of the CEBPB, MITF, FOXM1 among the top activated transcriptional regulators [Supplementary Figure 3B]. Pertinent to activation of the cWNT/β-catenin pathway, transcriptional regulators E2F1, E2F3, TCF7L2, and YAP were also predicted to be activated [Supplementary Figure 3B]. The DEGs also predicted suppression of transcriptional activities of NUPR1, CDKN2A, and KDM5B, among others, in the GoF myocytes [Supplementary Figure 3B]. Comparing the transcript levels between the LoF and GoF myocytes largely predicted dysregulation of the transcriptional regulators similar to that observed when comparing the GoF and WT myocytes [Supplementary Figure 3C].

**Figure 4.**
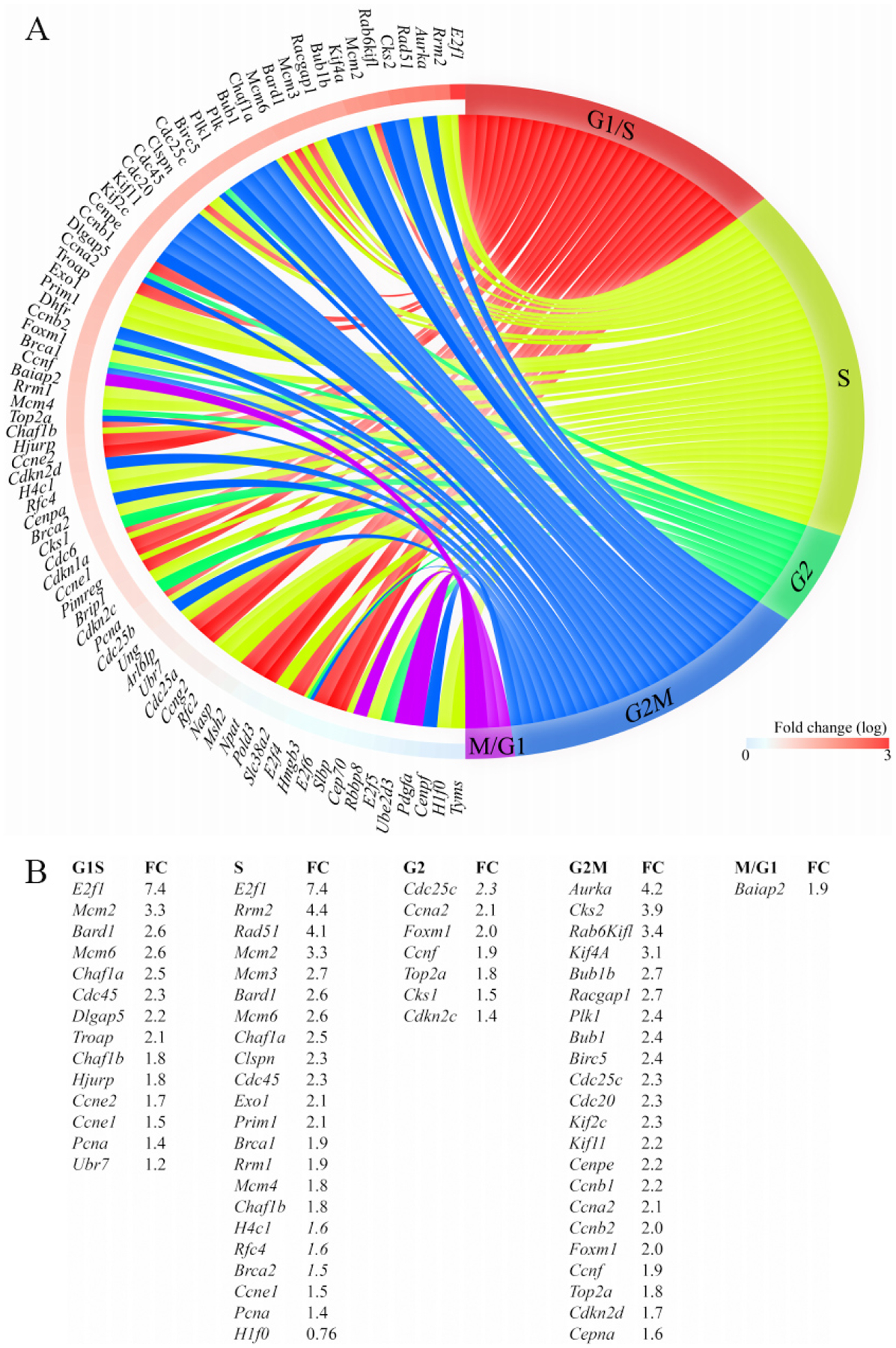
Increased transcript levels of genes involved in cell cycle progression in cardiac myocytes isolated from the *Myh6-Mcm:Ctnnb1*^GoF^ mice. (A) Circos plot depicting different phases of the cell cycle and the DEGs assigned to each phase. (B) Tabular listing of the DEGs in different phases of the cell cycle and changes in their expression levels, shown as fold change.

Given the prominent role of DEGs and the predicted transcriptional regulators, such as E2F1 and FOXM1, in the regulation of cell cycle progression, the DEGs were annotated to different phases of the cell cycle to gain insights into the effects of activation of the cWNT/β-catenin pathway on cardiac myocyte proliferation. Transcript levels of over 60 genes involved in the regulation of the cell cycle were increased in the GoF as compared to the WT myocytes [Figure 4A and B]. The vast majority of the upregulated genes have well-established functions in the S and G2M phases of the cell cycle. Notably, transcript levels of 22 genes known to regulate various stages of the M phase, including spindle assembly, centrosome duplication and separation, chromosome alignment, mitotic exit, and cytokinesis were increased [Figure 4A and B]. Thus, the data indicate that the activation of the cWNT/β-catenin pathway in the postmitotic myocytes is sufficient to induce the expression of a large number of genes involved in the advanced stages of the cell cycle, namely karyokinesis and cytokinesis.

### Effects of activation or suppression of the β-catenin on cardiac myocyte proliferation

**Figure 5.**
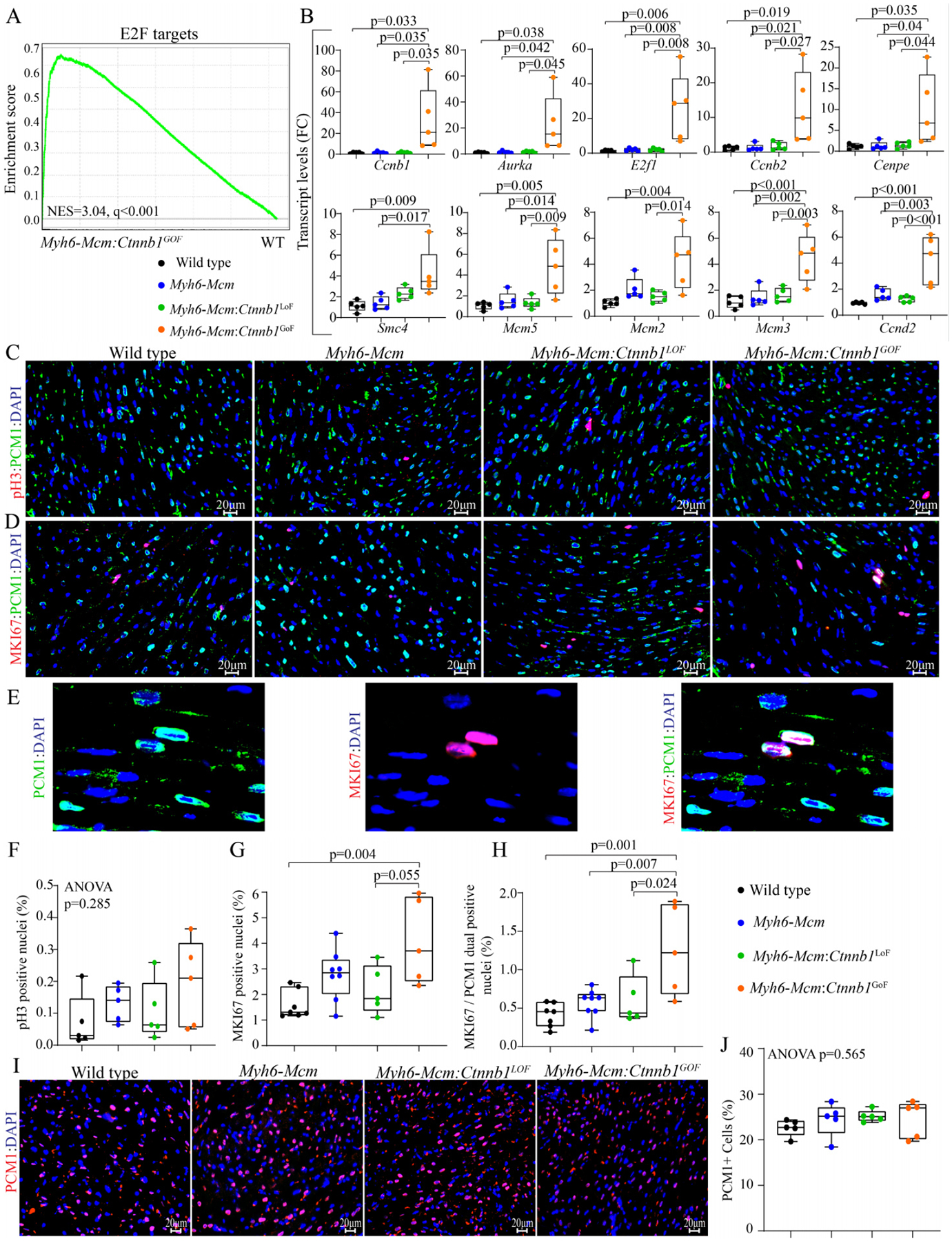
cWNT/β-catenin activation induces cell cycle reentry in postmitotic cardiac myocytes. (A) GSEA map depicting enrichment of the E2F targets in the DEGs in the β-catenin activated cardiac myocytes compared to the wild-type myocytes. (B) RT-PCR validation of the E2F target genes showing significant upregulation of cell proliferation markers in the β-catenin activated cardiac myocytes (*n* = 5). (C) Myocardial sections stained for phospho-histone 3 (pH3), a marker of proliferation, and PCM1, which tags cardiac myocytes in the heart in the experimental groups. (D) Representative immunofluorescence panels of thin myocardial sections stained for Ki-67, a marker of proliferation, and co-stained with PCM1, a marker for cardiac myocytes. (E) Enlarged images of *Myh6-Mcm:Ctnnb1*^GoF^ myocardial sections showing co-staining of Ki-67 (MKI67) and PCM1. (F-H) Quantitative data showing the comparison of the PH3 positive nuclei, Ki-67-stained nuclei, Ki-67, and PCM1 co-stained nuclei, as well as PCM1 positive nuclei between the wild type, *Myh6-Mcm*, *Myh6-Mcm:Ctnnb1*^LoF^, and *Myh6-Mcm:Ctnnb1*^GoF^ hearts (*n* = 5). (I) Representative immunofluorescence panels showing cardiac myocytes stained for PCM1 in the myocardial sections of WT, *Myh6-Mcm*, *Myh6-Mcm:Ctnnb1*^LoF^, and *Myh6-Mcm:Ctnnb1*^GoF^ mice. (J) Quantitative data showing the number of nuclei expressing PCM1 in the experimental groups, which was unchanged. RT-PCR: Reverse transcription-polymerase chain reaction; GSEA: Gene Set Enrichment Analysis; DEGs: differentially expressed genes; PCM1: pericentriolar material protein 1; WT: wild type; *Myh6-Mcm*: myosin heavy chain 6-MerCreMer.

Gene set enrichment analysis showed enrichment of the E2F target genes, which agrees with the pathway analysis of the RNA-Seq data [Figure 5A]. To test for corroboration of the findings, transcript levels of 10 genes involved in cell proliferation were analyzed by RT-PCR in independent samples. The findings were notable for markedly increased transcript levels of the selected genes in myocytes isolated from the *Myh6-Mcm*:*Ctnnb1*^GoF^ mice compared to the WT myocytes, corroborating the RNA-Seq findings [Figure 5B]. To further investigate cell cycle progression in myocytes, thin myocardial sections were co-stained for PCM1, a specific marker of cardiac myocytes in the heart, and phospho-H3 or MKI67 (Ki67), which are well-established markers of the cell cycle. The number of myocytes expressing pH3 was unchanged in experimental groups, whereas the number of myocytes co-expressing MKI67 and PCM1 was significantly increased in the *Myh6-Mcm*:*Ctnnb1*^GoF^ mouse hearts as compared to the WT or *Myh6-Mcm*:*Ctnnb1*^LoF^ genotypes [Figure 5C–H]. There were no differences in the percentages of nuclei expressing pH3 or MKI67 among the WT, *Myh6-Mcm*, and *Myh6-Mcm*:*Ctnnb1*^LoF^ genotypes [Figure 5C–H]. To determine whether cell cycle entry was associated with myocyte proliferation, the percentage of myocardial cells expressing PCM1, a surrogate marker for the number of myocytes in the heart, was determined in each experimental group, which was unchanged [Figure 5I and J].

### Effects of activation or suppression of the β-catenin on transcript levels of mitochondrial genes in cardiac myocytes

**Figure 6.**
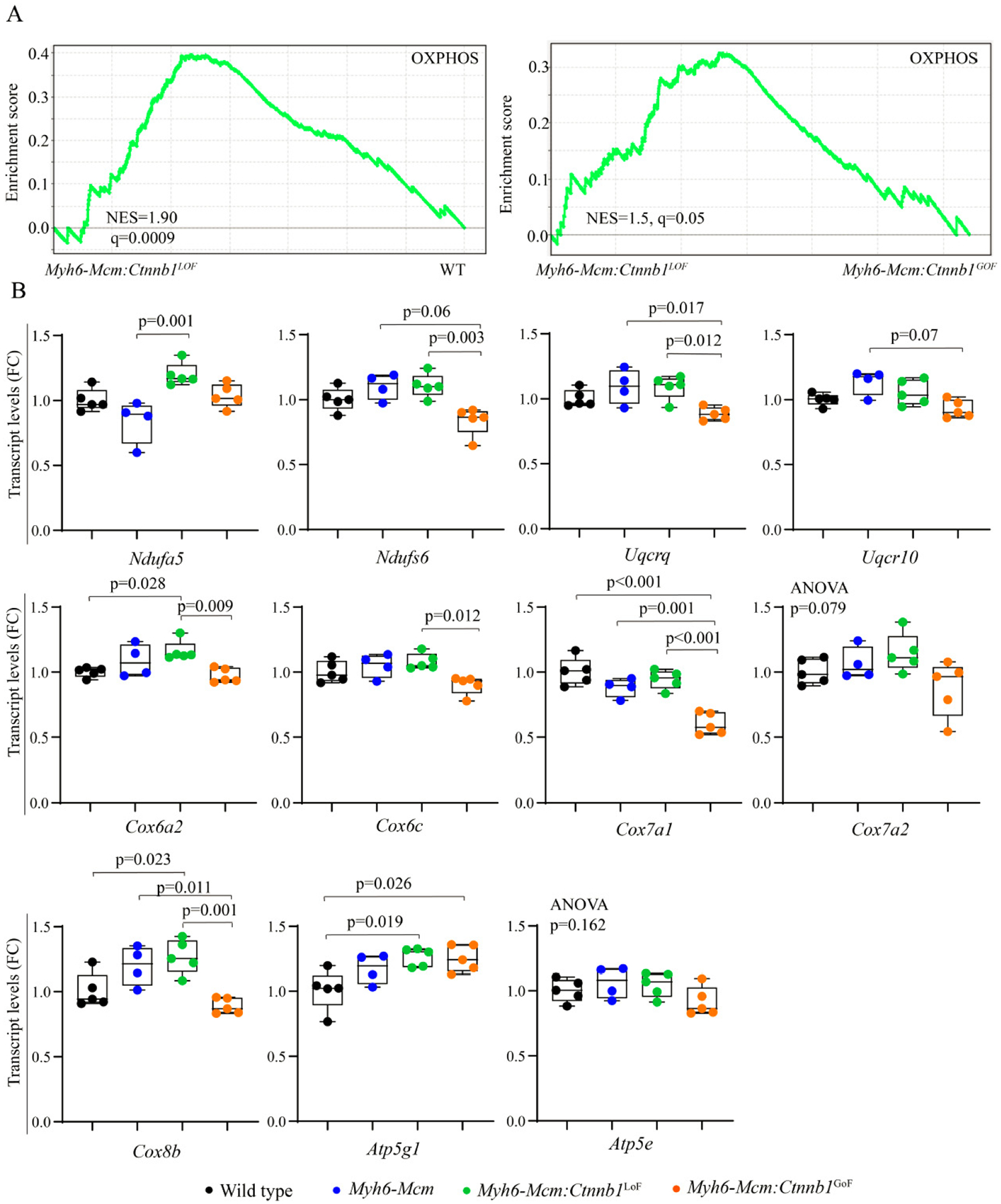
Suppression of β-catenin in cardiac myocytes induces genes involved in the mitochondrial OXPHOS pathway. (A) GSEA plots of the DEGs in cardiac myocytes from the *Myh6-Mcm:Ctnnb1*^LoF^ mouse (β-catenin suppression) showing activation of genes involved in the mitochondrial OXPHOS as compared to the wild type myocytes. A similar comparison between the *Myh6-Mcm:Ctnnb1*^LoF^ and *Myh6-Mcm:Ctnnb1*^GoF^ genotypes also shows activation of the OXPHOS pathway in the *Myh6-Mcm:Ctnnb1*^LoF^ mouse hearts. (B) RT-PCR validation of the transcript levels of genes involved in mitochondrial OXPHOS pathway in the *Myh6-Mcm:Ctnnb1*^LoF^ cardiac myocytes at 4 weeks of age (*n* = 4–5). GSEA: Gene Set Enrichment Analysis; DEGs: differentially expressed genes; OXPHOS: oxidative phosphorylation; *Myh6-Mcm*: myosin heavy chain 6-MerCreMer; RT-PCR: reverse transcription-polymerase chain reaction.

Bioinformatics analysis of the RNA-Seq data suggested activation of OXPHOS and TCA cycle in the *Myh6-Mcm*:*Ctnnb1*^LoF^ myocytes, as compared to the WT or the *Myh6-Mcm*:*Ctnnb1*^GoF^ myocytes [Figure 6A]. To test for corroboration of the findings, transcript levels of about a dozen genes coding for the proteins involved in OXPHOS were analyzed by RT-PCR in an independent set of samples. The findings were notable for a modest increase in the transcript levels in the *Myh6-Mcm*:*Ctnnb1*^LoF^ myocytes and, conversely, a modest decrease in the *Myh6-Mcm*:*Ctnnb1*^GoF^ myocytes [Figure 6B]. The findings, despite the modest changes, corroborate the RNA-Seq data suggesting activation of mitochondrial OXPHOS upon suppression of the cWNT/β-catenin pathway.

### Short-term phenotypic consequences of activation or suppression of the cWNT/β-catenin pathway in the heart

Given the focus of the study on identifying the transcriptomic changes imparted by the cWNT/β-catenin pathway in the heart, the additional phenotypic data were analyzed at 4 weeks of age, coinciding with the timing of RNA-Sequencing. Long-term effects of activation or suppression of the β-catenin have been studied before and, therefore, were not pursued^[6]^.

#### Survival rates

LoF and GoF of β-catenin had no significant effect on survival, as the survival rates in the *Myh6-Mcm*:*Ctnnb1*^LoF^ and *Myh6-Mcm*:*Ctnnb1*^GoF^ mice, injected with tamoxifen, were similar to the corresponding WT and *Myh6*-*MCM* mice; the latter also were similarly injected with tamoxifen up to 6 months of age (the last time point analyzed).

#### Cardiac size and function

Effects of suppression or activation of the β-catenin on cardiac size and function were determined at 4 weeks of age by echocardiography. Activation or suppression of the cWNT in cardiac myocytes had no significant effect on the LVEDD, LVESD, or LVEF [Table 1]. However, when indexed to body weight, LVEDD and LVM were modestly increased in the *Myh6-Mcm:Ctnnb1*^LoF^ and *Myh6-Mcm:Ctnnb1*^GoF^ mice. Detailed echocardiographic data are presented in Table 1.

#### Heart and cardiac myocyte size

**Figure 7.**
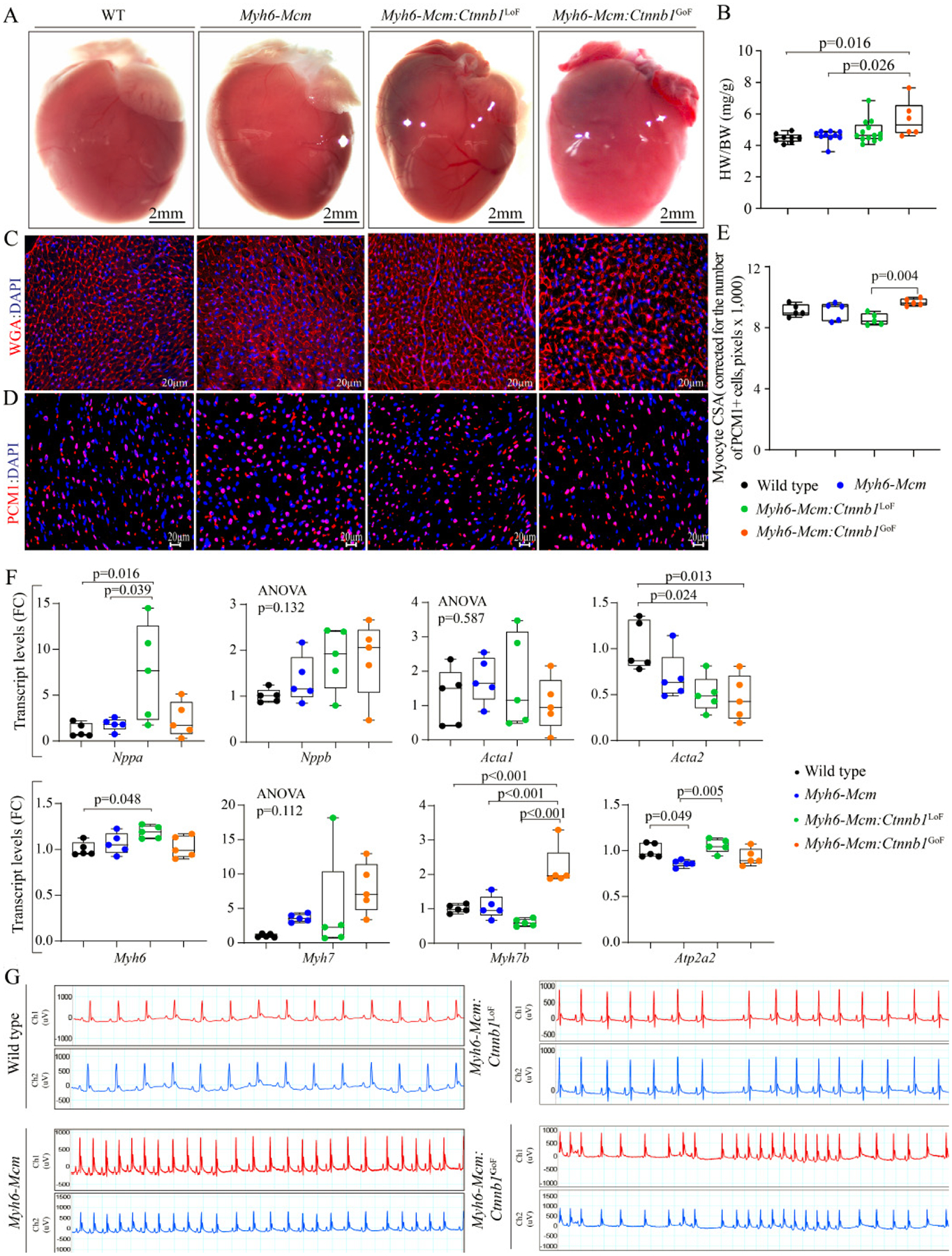
Effect of suppression or activation of β-catenin in cardiac myocytes on cardiac size and rhythm. (A) Gross morphology of the mouse hearts. (B) Heart weight/body weight ratio in the experimental groups at 4 weeks of age. (C) Representative immunofluorescence panels of myocardial sections stained for wheat germ agglutinin (WGA) and counterstained with DAPI. (D) Panels showing thin myocardial sections stained for PCM1 to identify cardiac myocytes. (E) Quantitation of myocyte cross-sectional area (CSA), corrected for the number of PCM1-stained cardiac myocytes, in the experimental groups showing a significant increase in the myocyte CSA in the *Myh6-Mcm:Ctnnb1*^GoF^ (GoF) hearts (*n* = 5). (F) Transcript levels of genes involved in determining cardiac size and function in wild type, *Myh6-Mcm:Ctnnb1*^LoF^ and *Myh6-Mcm:Ctnnb1*^GoF^ myocytes, quantitated by RT-PCR (*n* = 5). (G) Representative 2-lead rhythm monitoring strips in the 4 weeks old mice obtained from the surface electrocardiographic recordings in the experimental groups. PCM1: Pericentriolar material protein 1; *Myh6-Mcm*: myosin heavy chain 6-MerCreMer; RT-PCR: reverse transcription-polymerase chain reaction.

To determine whether activation or suppression of the β-catenin in quiescent cardiac myocytes affected cardiac mass, heart weight to body weight ratio (HW/BW) was determined in the experimental groups. Only GoF of the β-catenin, i.e., the *Myh6-Mcm:Ctnnb1*^GoF^ mice, showed a modest increase in the HW/BW ratio compared to the WT or *Myh6-Mcm* mice, the latter also treated with tamoxifen [Figure 7A and B]. To further assess the increased cardiac mass, myocyte CSA was calculated upon staining of the thin myocardial sections with WGA to define the interstitium and PCM1 to identify cardiac myocytes [Figure 7C]^[24,30,31,34]^. The myocyte cross-sectional area was increased in the *Myh6-Mcm:Ctnnb1*^GoF^ as compared to the *Myh6-Mcm:Ctnnb1*^LoF^ mice but was not different from that in the WT or *Myh6-Mcm* mice [Figure 7D and E].

To further assess the effects of the GoF and LoF of β-catenin on cardiac size and function, transcript levels of selected markers were assessed by RT-PCR. There were modest but significant changes in the transcript levels of several markers except for the *Myh7b*, whose transcript levels were markedly increased in myocytes isolated from the *Myh6-Mcm:Ctnnb1*^GoF^ mice [Figure 7F].

#### Cardiac arrhythmias

All mice exhibited normal sinus rhythm, sinus arrhythmias, sinus bradycardia, or sinus tachycardia during 2-lead surface electrocardiography recording for 1 h [Figure 7G]. Rare ventricular premature beats were detected in mice in all groups. No significant supraventricular or ventricular arrhythmias were recorded in the *Myh6-MCM:Ctnnb1*^LoF^ and *Myh6-MCM:Ctnnb1*^GoF^ mice [Figure 7G].

#### Myocardial fibrosis

**Figure 8.**
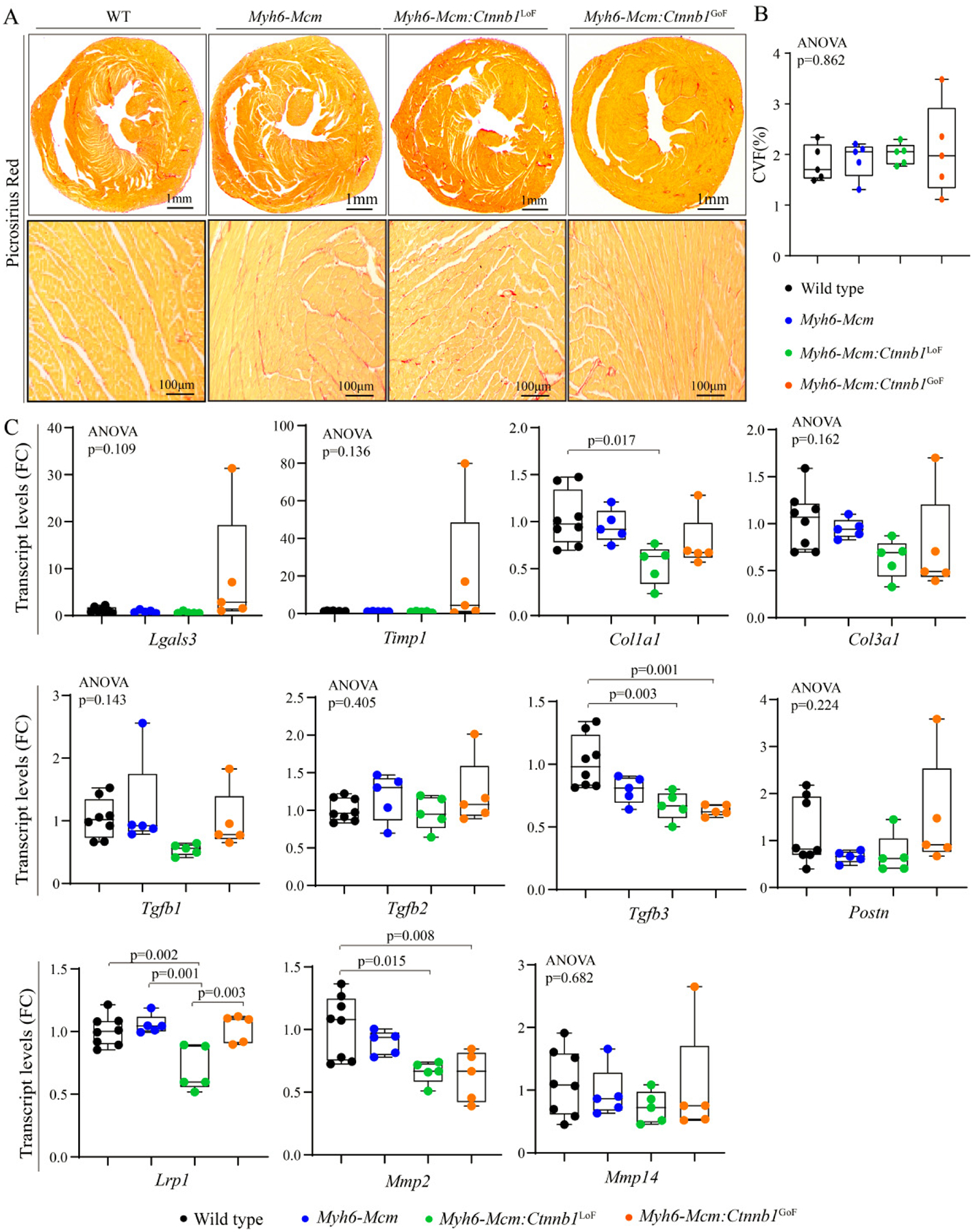
Effects of suppression or activation of the β-catenin on myocardial fibrosis in 4-week-old mice. (A) Picrosirius red-stained thin myocardial sections in 4 weeks old wild type, *Myh6-Mcm*, β-catenin LoF and GoF hearts showing no change in myocardial fibrosis. (B) Quantitation of myocardial fibrosis presented as % collagen volume fraction (CVF) in the experimental groups (*n* = 5). (C) RT-PCR data showing transcript levels of genes involved in myocardial fibrosis in the β-catenin LoF and GoF hearts along with the control hearts (*n* = 5). *Myh6-Mcm*: Myosin heavy chain 6-MerCreMer; RT-PCR: reverse transcription-polymerase chain reaction; GoF: gain-of-function; LoF: loss-of-function.

To determine the extent of myocardial fibrosis upon suppression or activation of the cWNT/β-catenin pathway in cardiac myocytes, myocardial CVF was determined in the experimental groups upon staining thin myocardial sections with picrosirius red [Figure 8A]. Neither LoF nor GoF of the β-catenin affected the CVF [Figure 8A and B]. To further assess the effects on myocardial fibrosis, transcript levels of several genes involved in myocardial fibrosis were quantified by RT-PCR in independent samples. There were modest changes in the transcript levels of *Col1a1*, *Tgfb3*, *Lrp1*, and *Mmp2* [Figure 8C].

#### Myocardial apoptosis

**Figure 9.**
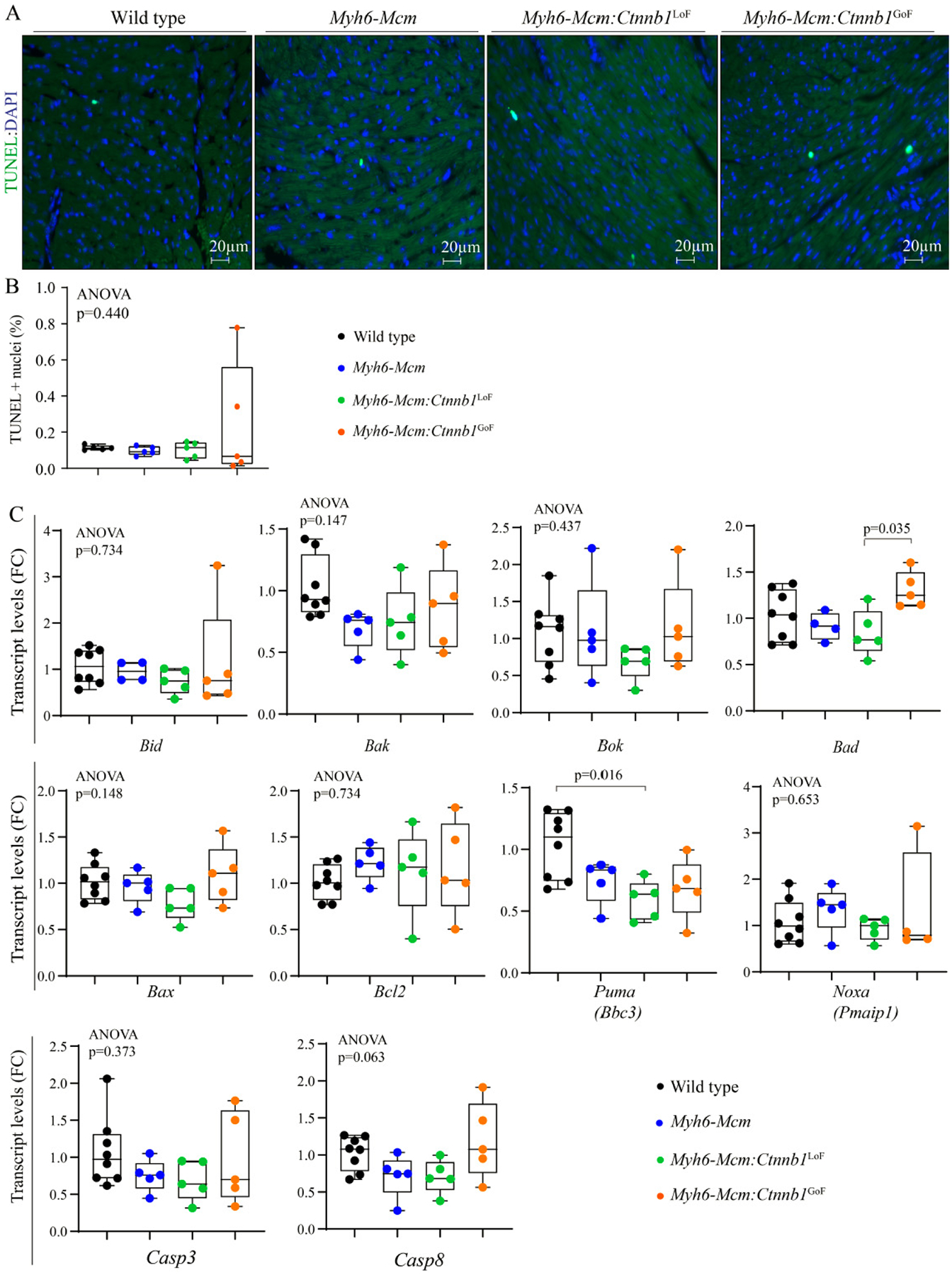
Effect of suppression or activation of the β-catenin in the postmitotic cardiac myocytes on myocardial apoptosis at 4 weeks of age. (A) Representative images of Terminal deoxynucleotidyl transferase dUTP nick end labeling (TUNEL) stained thin myocardial sections at 4 weeks of age in WT, *Myh6-Mcm*, *Myh6-Mcm:Ctnnb1*^LoF^, and *Myh6-Mcm:Ctnnb1*^GoF^ hearts showing no difference in the number of TUNEL-stained cells. (B) Quantitative data showing the number of TUNEL-positive nuclei in the WT, *Myh6-Mcm*, *Myh6-Mcm:Ctnnb1*^LoF^, and *Myh6-Mcm:Ctnnb1*^GoF^ hearts (*n* = 5). (C) RT-PCR data showing transcript levels of genes involved in regulating myocardial apoptosis in the four experimental groups (*n* = 4–5). WT: Wild type; *Myh6-Mcm*: myosin heavy chain 6-MerCreMer; RT-PCR: reverse transcription-polymerase chain reaction.

The number of apoptotic cells in the myocardium was determined upon staining of the thin myocardial sections with the TUNEL assay. Neither LoF nor GoF of the β-catenin affected the number of TUNEL stained myocardial cells [Figure 9A and B]. Likewise, the transcript levels of several genes involved in apoptosis were quantified in independent RNA samples, which showed no consistent changes to indicate increased pro-apoptotic gene expression [Figure 9C].

#### Myocardial adipogenesis

**Figure 10.**
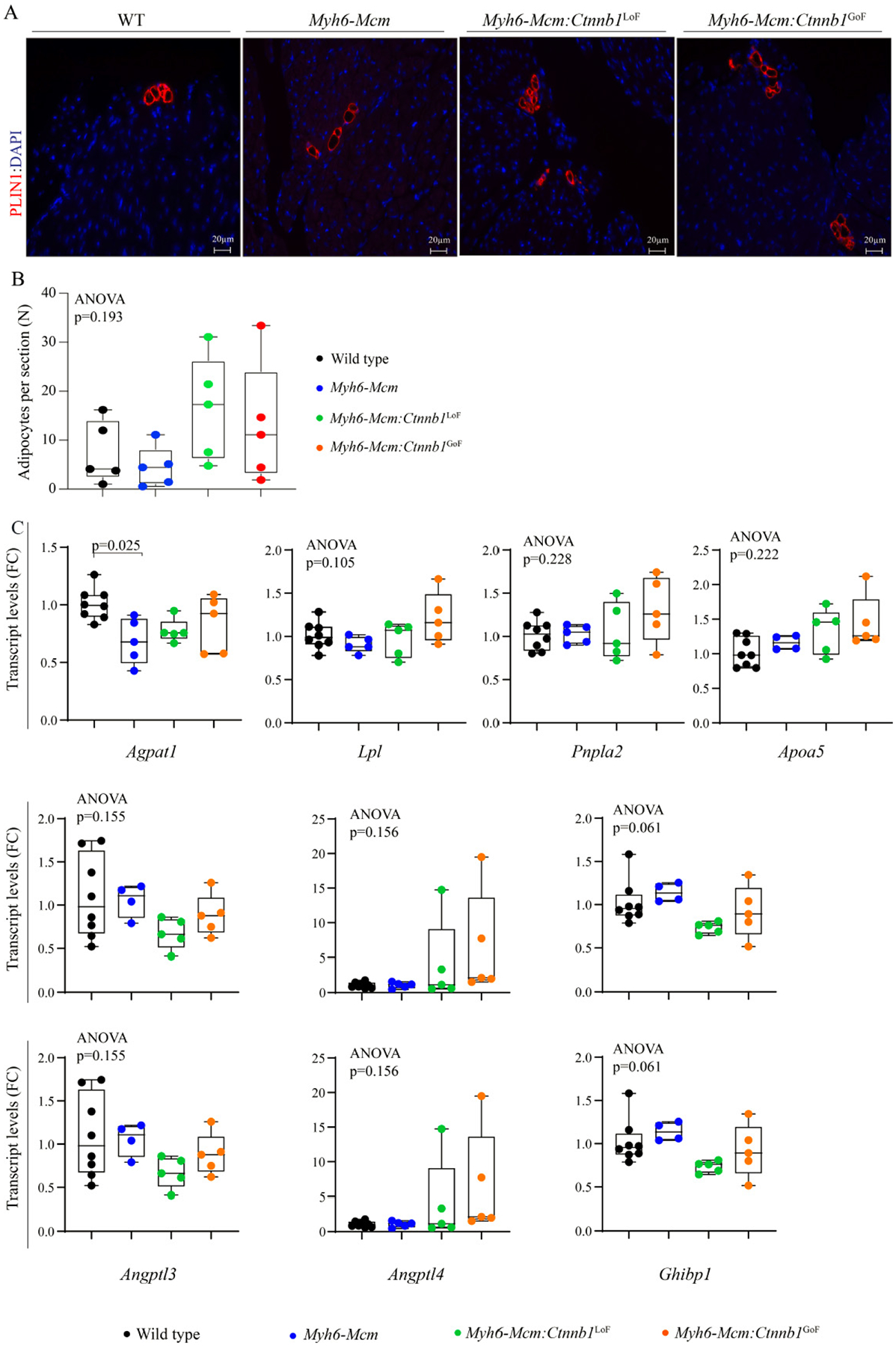
Myocardial adipogenesis upon suppression or activation of the β-catenin in the postmitotic cardiac myocytes at 4 weeks of age. (A) Representative immunofluorescence images of Perilipin 1 stained thin myocardial sections in the WT, *Myh6-Mcm*, *Myh6-Mcm:Ctnnb1*^LoF^, and *Myh6-Mcm:Ctnnb1*^GoF^ hearts at 4 weeks of age. (B) Quantitative data showing the number of adipocytes in the myocardium following suppression or activation of the cWNT/β-catenin pathway in the postmitotic cardiac myocytes (*n* = 5). (C) RT-PCR data showing transcript levels of selected genes involved in adipogenesis in the hearts of 4 weeks old WT, *Myh6-Mcm*, *Myh6-Mcm:Ctnnb1*^LoF^, and *Myh6-Mcm:Ctnnb1*^GoF^ mice (*n* = 4–5). WT: Wild type; *Myh6-Mcm*: myosin heavy chain 6-MerCreMer; RT-PCR: reverse transcription-polymerase chain reaction.

To determine the effects of suppression or activation of the β-catenin on myocardial adipogenesis, the number of mature adipocytes in the myocardium was determined upon staining the myocardial sections with an antibody against PLIN1, which tags the triglyceride-rich adipocytes^[35]^. Overall, at the young age of 4 weeks, the number of adipocytes in the heart were similar among the experimental groups as the LoF or GoF of the β-catenin had no discernible effect on the number of mature adipocytes in the heart at the young age of 4 weeks [Figure 10A and B]. Furthermore, quantification of the transcript levels of several genes involved in adipogenesis by RT-PCR in independent samples did not show significant changes among the experimental groups [Figure 10C].

## DISCUSSION

The cWNT/β-catenin pathway is involved in cardiac myocyte proliferation during embryogenesis and in the pathogenesis of cardiovascular diseases, including cardiomyopathies^[10]^. Despite the interest in the role of the cWNT/β-catenin pathway in cardiovascular pathology, its effects on gene expression in quiescent cardiac myocytes have remained unknown. Through a set of GoF and LoF studies, we show that genetic activation or suppression of the β-catenin led to altered expression of over 1500 genes in cardiac myocytes. GoF of the β-catenin had the most remarkable effect on gene expression and strongly provoked cell cycle reentry of the quiescent cardiac myocytes through activation of the E2F pathway and others, but did not result in proliferation, as the number of myocytes remained unchanged. The LoF of the β-catenin affected a smaller number of genes and modestly activated the expression of genes involved in OXPHOS. The smaller number of the DEGs upon LoF, as opposed to the GoF in cardiac myocytes, is in accord with the relatively dormant activity of the cWNT/β-catenin pathway in quiescent cardiac myocytes. Collectively, the data emphasize the role of the cWNT/β-catenin pathway in cardiac myocyte cell cycle reentry, albeit without myocyte proliferation, and also suggest a potential role for this pathway in mitochondrial OXPHOS.

The fidelity of the genetic LoF and GoF mouse models was established across multiple platforms, including immunoblotting, RT-PCR, and RNA-Seq approaches. Genes regulated by the cWNT/β-catenin pathway were identified in the absence of cardiac dysfunction and altered myocardial architecture, and therefore, were likely primary effects of the cWNT/β-catenin and largely free of the confounding effects of cardiac dysfunction or fibrosis. In addition, the RNA-Seq experiments benefited from robust quality control and a high read number, enabling identification of a large number of DEGs upon activation or suppression of the β-catenin in cardiac myocytes. Furthermore, the phenotypic consequences of genetic activation or suppression of the β-catenin were concordant across cardiac phenotypes in the *Myh6-Mcm:Ctnnb1*^LoF^ and *Myh6-Mcm:Ctnnb1*^GoF^ mice and were corroborated using complementary methods.

Activation of the β-catenin upon deletion of the GSK3β phosphorylation sites provoked activation of the E2F1 and 3 transcription factors and reentry of the postnatal cardiac myocytes into the cell cycle. However, it was insufficient to induce the proliferation of quiescent cardiac myocytes. This is in contrast to some other cell types in which activation of the cWNT/β-catenin leads to proliferation. The mechanism(s) necessary to complete the cell cycle, including karyokinesis and cytokinesis of postnatal cardiac myocytes, were not explored in the present study. In contrast, suppression of the β-catenin upon genetic deletion of the transactivation domain of β-catenin led to a modest increase in the transcript levels of genes involved in OXPHOPS in cardiac myocytes, albeit the changes were modest and mostly not significant. It remains to be seen whether in pathological conditions such as heart failure whereby OXPHOS is suppressed, suppression of the cWNT/β-catenin pathway could attenuate or rescue the heart failure phenotype by upregulating mitochondrial OXPHOS. This notion is in agreement with the results of a recent study showing that pharmacological inhibition of the cWNT by targeting porcupine partially rescues cardiac dysfunction and fibrosis in a mouse model of arrhythmogenic cardiomyopathy^[4]^.

The study has several limitations. The study was a short-term study designed to identify the targets of the cWNT/β-catenin pathway in postnatal cardiac myocytes. Consequently, it was not designed to study the effects of the activation or suppression of the cWNT/β-catenin pathway on long-term cardiac structure and function. Previous studies have investigated the long-term effects of activation or suppression of the cWNT/β-catenin in the heart^[6,7,36]^. Nevertheless, the long-term effects of the transcriptional changes induced upon activation or suppression of the cWNT/β-catenin pathway merits further investigation. In addition, cell cycle progression in the postnatal cardiac myocytes upon activation of the cWNT pathway was not investigated in great detail, partly because the number of myocytes, the end product of cell cycle reentry, was unchanged. Furthermore, mitochondrial OXPHOS was analyzed only at the transcript levels and was not pursued further, given the modest nature of the changes. Finally, bioinformatics analysis of the transcripts predicted dysregulation of several biological pathways and regulators of gene expression, which should be considered provisional, hypothesis-generating, and exploratory, requiring validation through alternative methods.

In conclusion, the data indicate that genetic activation or suppression of the cWNT/β-catenin in postmitotic cardiac myocytes in mice leads to altered transcript levels of more than 1500 genes, particularly those involved in cell cycle regulation and mitochondrial OXPHOS. The findings upon activation of the β-catenin set the stage for delineating the mechanisms involved in myocyte proliferation. In contrast, the data suggesting activation of OXPHOS upon suppression of the β-catenin, if validated in pathological states, would suggest potential salutary effects of the suppression of the cWNT pathway in attenuating and rescuing the cardiovascular conditions whereby OXPHOS is suppressed, such as in heart failure.

## Supplementary Material

Supplementary Material

## Figures and Tables

**Table 1.** Echocardiographic indices of cardiac size and function

	WT	*Myh6-Mcm*	*Myh6-Mcm:Ctnnb1LoF*	*Myh6-Mcm:Ctnnb1GoF*	One way ANOVA
*n*	9	10	16	12	NA
M/F	4/5	5/5	8/8	6/6	0.993^$^
Age (days)	28.22 ± 0.44	28.70 ± 0.67	28.63 ± 0.81	28.50 ± 0.67	0.444
Body weight (g)	16.24 ± 1.82	15.64 ± 1.26	13.61 ± 1.83**^,#^	13.96 ± 1.61*	0.0008
HR (bpm)	660.04 ± 29.14	674.18 ± 38.22	638.19 ± 36.56	632.43 ± 36.12	0.0284
AWT (mm)	0.36 ± 0.02	0.34 ± 0.02	0.34 ± 0.02	0.34 ± 0.02	0.0396
LVPWT (mm)	0.36 ± 0.02	0.34 ± 0.02	0.33 ± 0.02	0.33 ± 0.02	0.0488
LVEDD (mm)	2.81 ± 0.30	2.96 ± 0.19	2.90 ± 0.28	3.02 ± 0.32	0.3453
LVEDDI (mm/g)	0.17 ± 0.02	0.19 ± 0.02	0.22 ± 0.02***^,#^	0.22 ± 0.02***^,#^	< 0.0001
LVESD (mm)	1.52 ± 0.15	1.60 ± 0.12	1.57 ± 0.17	1.69 ± 0.17	0.0805
LVESDI (mm/g)	0.09 ± 01	0.10 ± 0.01	0.12 ± 0.01***^,#^	0.12 ± 0.01***^,#^	< 0.0001
LVFS (%)	45.73 ± 2.29	46.03 ± 2.45	46.04 ± 2.60	43.98 ± 1.78	0.1022
LV mass (mg)	18.27 ± 4.68	18.55 ± 1.95	17.82 ± 4.05	19.06 ± 4.10	0.8641
LVMI (mg/g)	1.12 ± 0.24	1.19 ± 0.12	1.30 ± 0.18	1.36 ± 0.22*	0.0314

Data in the WT and *Myh6-Mcm* groups are from Rouhi *et al*.^[33]^.

$ Denotes *P*-value obtained by Chi-square test.

* Denotes *P* < 0.05 compared to wild type.

** Denotes *P* < 0.01 compared to wild type.

*** Denotes *P* < 0.005 compared to wild type.

# Denotes *P* < 0.05compared to *Myh6-Mcm* group.

*Myh6-Mcm*: Myosin heavy chain 6-MerCreMer; F/M: female/male; g: grams; HR: heart rate; bpm: beats per min; AWT: anterior wall thickness; LVPWT: left ventricular posterior wall thickness; LVEDD: left ventricular end-diastolic diameter; LVEDDI: LVEDD indexed to body weight; LVESD: left ventricular end-systolic diameter; LVESDI: LVESD indexed to body weight; LVFS: left ventricular fractional shortening; LVM: left ventricular mass; LVMI: LVM indexed to body weight.

## Data Availability

RNA-Seq data have been submitted to GEO and available to the public upon release (GSE180972).
