# Supplementary material for: The WNT/β-catenin pathway regulates expression of the genes involved in cell cycle progression and mitochondrial oxidative phosphorylation in the postmitotic cardiac myocytes
Source: J Cardiovasc Aging. Author manuscript; Available in PMC 2022 Feb 25. (PMC8874274; doi:10.20517/jca.2021.35)
Supplement: Supplementary Material [file NIHMS1775796-supplement-Supplementary_Material.pdf]

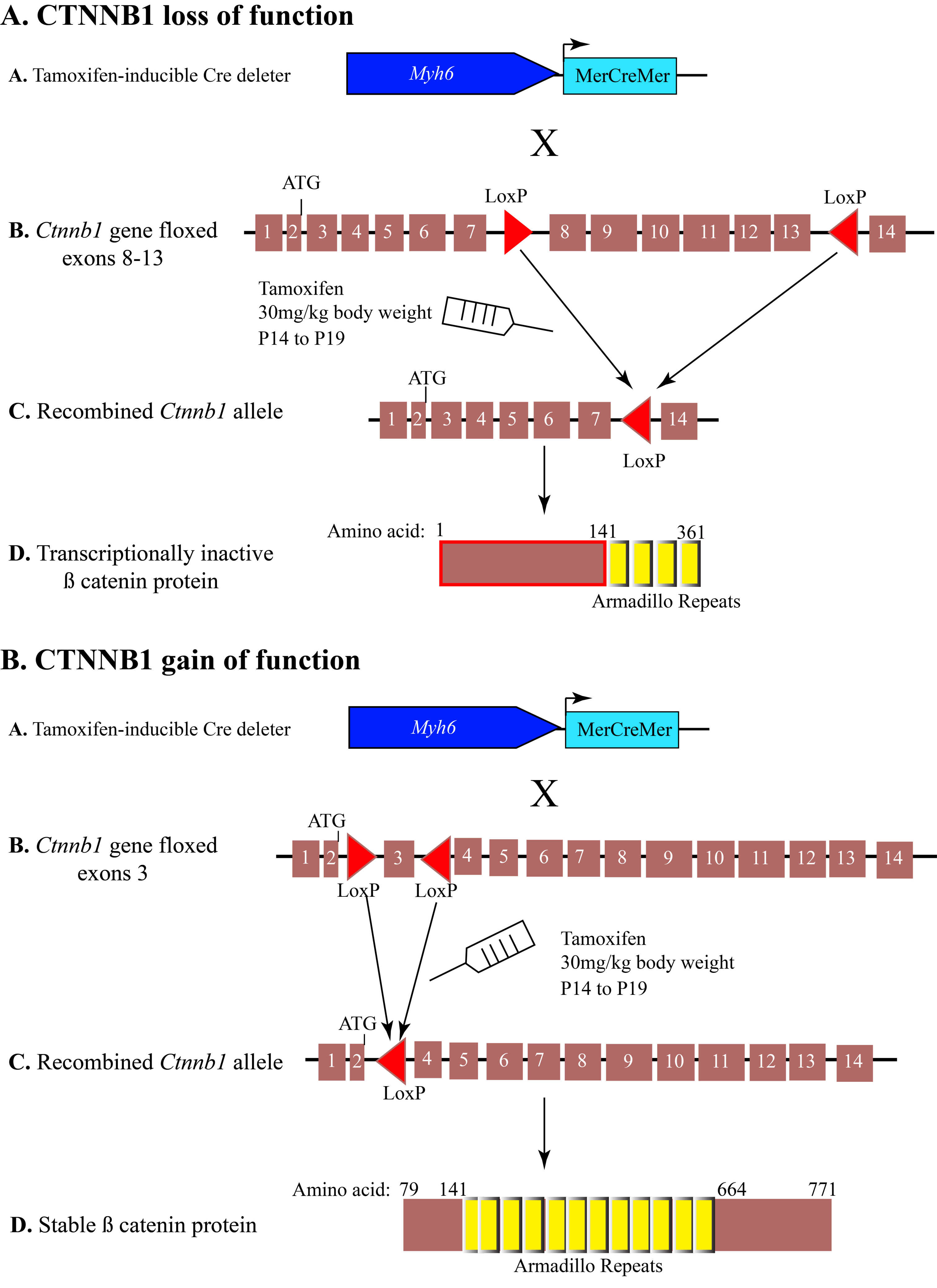

Online Figure 1. Schematic illustration of loss of function and gain of function ß catenin mice using *Myh6-MerCreMer* and *Ctnnb1* floxed alleles

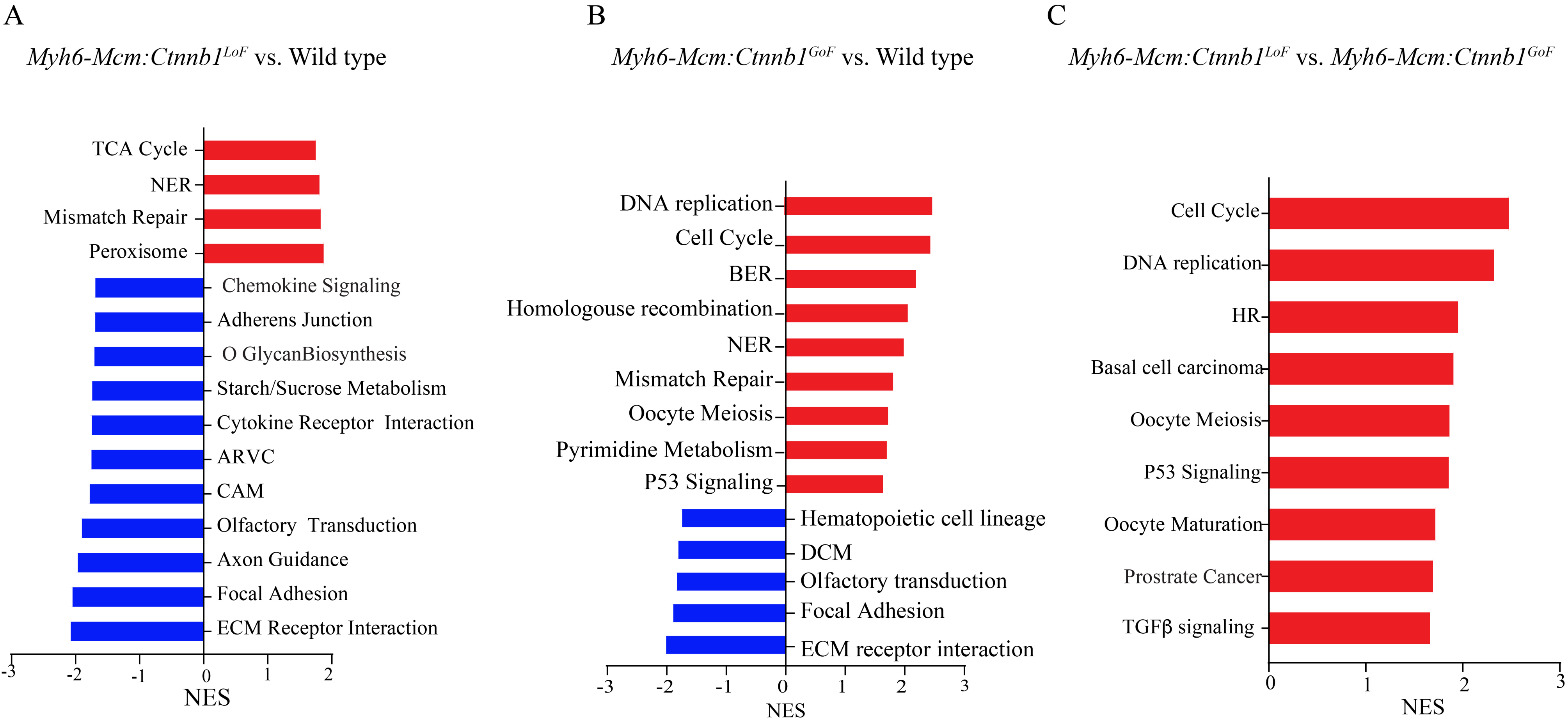

Online Figure 2. Biological pathways predicted to be dsyregulated in the myocytes isolated from the *Myh6-Mcm:Ctnnb1*^{LoF} (A), *Myh6-Mcm:Ctnnb1*^{GoF} (B) compared to myocytes isolated from the wild type mice. Panel C depicts the predicted biological pathways dysregulated when comparing the DEGs in the myocytes from the *Myh6-Mcm:Ctnnb1*^{LoF} and *Myh6-Mcm:Ctnnb1*^{GoF} mice. The NES values are depicted only for pathways that had a q-value of <0.05.

Abbreviations:

NES: Normalized enrichment score.
TCA: tricarboxylic acid
NER: Nucleotide excision repair
ARVC: Arrhythmogenic right ventricular cardiomyopathy
CAM: Crassulacean acid metabolism
ECM: Extracellular matrix
BER: Base excision repair
DCM: Dilated cardiomyopathy
HR: Homologous recombination
TGFβ: Transforming growth factor beta

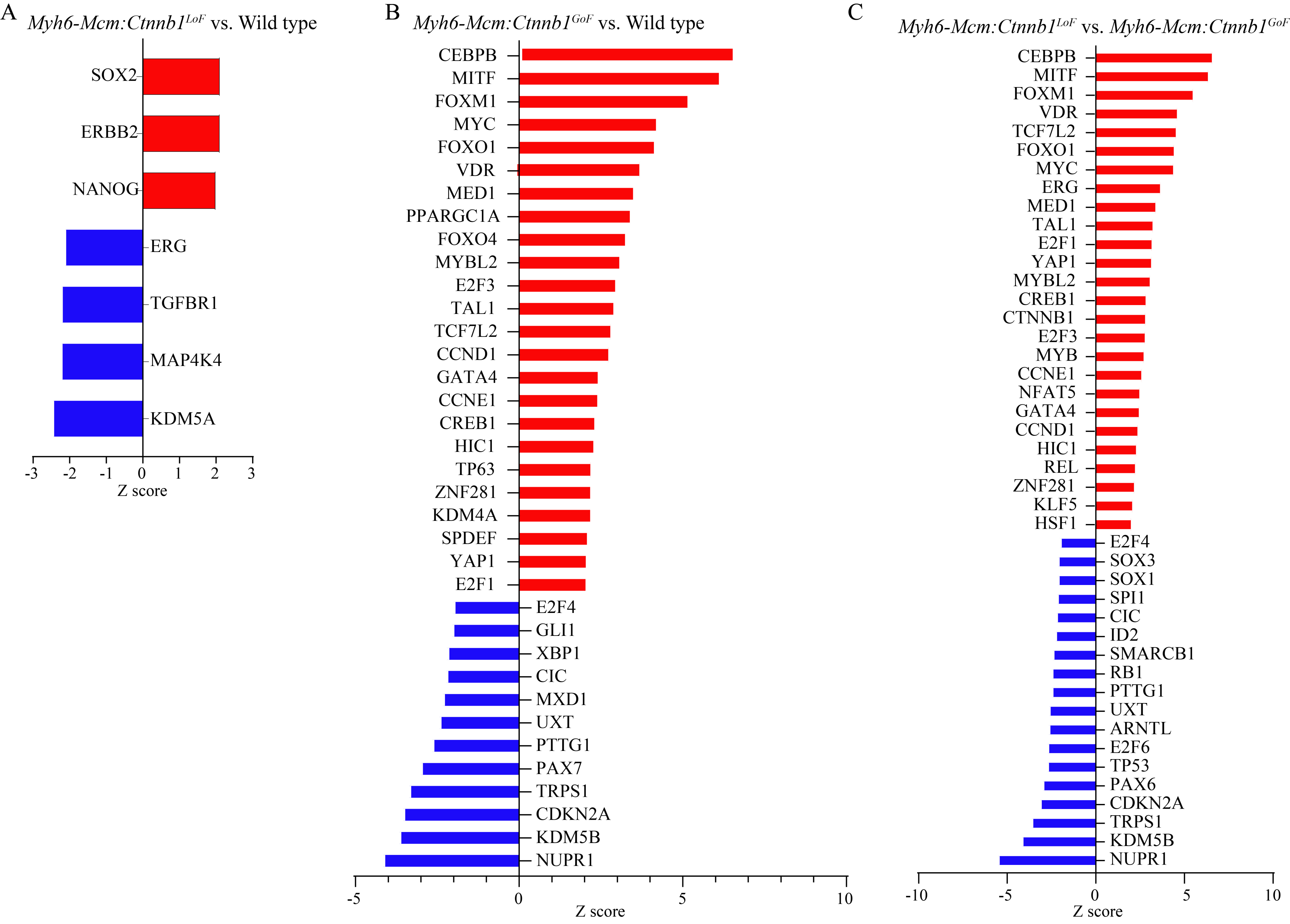

Online Figure 3. Transcriptional regulators of gene expression predicted to be dsyregulated in the myocytes isolated from the *Myh6-Mcm:Ctnnb1*^{LoF} (A) *Myh6-Mcm:Ctnnb1*^{GoF} (B) compared to myocytes isolated from the wild type mice. Panel C depicts predicted transcriptional regulators of the DEGs the myocytes from the *Myh6-Mcm:Ctnnb1*^{LoF} and *Myh6-Mcm:Ctnnb1*^{GoF} mice.

Online Table 1: Oligonucleotide Primers, Antibodies, and TaqMan probes

Genotyping primers:

Gene	Forward sequence	Reverse sequence
Cre recombinase	TCTATTGCACACAGCAATCCA	CCAGCATTGTGAGAACAAGG
Ctnnb1(Ex3)	AGACTGCCTTGGGAAAAGCGC	TGGATGGGATCTGCATGCCCTCATCTA
Ctnnb1(Ex8-13)	TGCAGCTTACCTGACTCCTG	CCTGTTAGCCCTCATGGTGT

List of antibodies

Antibodies	Concentration	Supplier	Catalog number
Anti-mouse IgG HRP linked antibody	1:4000 (IB)	Cell Signaling Technology	7076
Anti-Rabbit IgG HRP linked antibody	1:2000 (IB)	Cell Signaling Technology	7074
Donkey anti Rat Alexa Fluor 594	1:1000 (IF)	Invitrogen	A11007
Goat anti Mouse Alexa Fluor594	1:1000 (IF)	Invitrogen	A11005
Goat anti Rabbit Alexa Fluor 488	1:1000 (IF)	Invitrogen	A11012
GAPDH	1:1000 (IB)	Cell Signaling Technology	2118
Vinculin	1:10000 (IB)	Abcam	Ab129002
CTNNB1 (C-terminal)	1:1000 (IB)<br>1:100 (IF)	Cell Signaling Technology	8480S
CTNNB1 (N-terminal)	1:1000 (IB)	Cell Signaling Technology	9562S
CTNNB1	1:50 (IF)	Cell Signaling Technology	9561S
Ki-67	1:250 (IF)	Abcam	ab15580
Phospho Histone H3	1:1000 (IF)	Abcam	ab10543
PCM1	1:1000 (IF)	Sigma	HPA023370
PCM1	1:50 (IF)	Santa Cruz	Sc-398365
PLIN	1:100 (IF)	Cell signaling	9349

Oligonucleotide primers used in qPCR reactions

Name	Sequence
Gapdh	Forward: AACTTTGGCATTGTGGAAGG<br>Reverse: GGATGCAGGGATGATGTTCT
Ctnnb1 GOF (ex3)	Forward: AGTCCTTCACGCAAGAGCAA<br>Reverse: CAAACTGCGTGGATGGGATC
Ctnnb1 GOF (ex3-4)	Forward: TTCCCAGTCCTTCACGCAAG<br>Reverse: TGCCCTCATCTAGCGTCTCA

Ctnnb1 LOF (ex9)	Forward: TCTTGTACGCACCGTCCTTC<br>Reverse: CCATAATGAAGGCGAACGGC
Ctnnb1 LOF (ex12-13)	Forward: ACTCCAGGAATGAAGGCGTG<br>Reverse: TGGTCAGCTCGACTGAAAGC
Ccnb1	Forward: GTGCCGCCTGTACATAGGAT<br>Reverse: TCGACAACTTCCGTTAGCCT
E2f1	Forward: CCACGAGGCCCTTGACTATC<br>Reverse: ACACCCTGAATCCCTAGGCT
Aurka	Forward: GAGCTACCGGGATCGCTTG<br>Reverse: CCGAAGGGAACAGTGGTCTT
Ccnb2	Forward: CTATCCGGCGGGCAGTTTTA<br>Reverse: GGCAGAGCTGGTACTTTGGT
Cenpe	Forward: AAGTTCGGAGAGAGTGCAGC<br>Reverse: TTCCTCGCTTAGGAGCGTTG
Mcm5	Forward: CGCCGCTGTGAGGTACA<br>Reverse: TCAGGAACTCCTTGAATCGCC
Smc4	Forward: CAGTCGTAACTTGGCCCTCG<br>Reverse: TTCTGGGACCCGCGAATTT
Mcm2	Forward: CTCCAAGGCTGGCATCGTTA<br>Reverse: CAAAGCGGGAAATGATGGGC
Mcm3	Forward: GGGCACAGTAGTGCTGGATG<br>Reverse: TCCCGAACCTTGTTCTGGTAA
Ccnd2	Forward: ATTCAGCCAAAGGAAGGAGGTAAG<br>Reverse: ACCACTCGGTCCCGACTGTAA
Cox7a1	Forward: TGCCAGGATCCGGAGTCTTAGAA<br>Reverse: GGTCATTGTCGGCCTGGAAG
Atp5e	Forward: TGGACTCAGCTACATCCGGT<br>Reverse: CGTTCGCTTTGAACTCGGTC
Cox6a2	Forward: GCCCAGAGTTCATCCCGTAT<br>Reverse: AGGATTGACGTGGGGATTGT
Atp5g1	Forward: CGCTCAGACCAAGGGCTAAA<br>Reverse: CCCTGGTACAGGAGCGAATC
Cox7a2	Forward: AGAGGACCATCAGCACCACT<br>Reverse: CATCAGATGCCCCGCCTTTC
Cox8b	Forward: GCCAGCCAAAACTCCCACTTC<br>Reverse: GCTCTCCAAGTGGGCTAAGA
Cox6c	Forward: GGCTGGTATCTTTCAGAGTGC<br>Reverse: TAGTTCAGGAGCGCAGGTCA
Ndufa5	Forward: AAGCTGGATATGGTCAAGGCG<br>Reverse: CTCTTCCACTTCACCACCCT
Ndufs6	Forward: TTTCGGGGTTCAAGTGTCGC<br>Reverse: CGAACCCTCCTGTAGTCTTT
Uqcr10	Forward: CGCAGAACTTCCACCTTTGC<br>Reverse: CCACAGTTTCCCCTCGTTGA
Uqcrq	Forward: GCCTTCCCAAGCTATTTCAGCA<br>Reverse: ACCTGGTTGCCCCATGTGTAG
Vcl	Forward: GGTCTAGCAAGGGCAATGAC<br>Reverse: TGAATAAGTGCCCGCTTGGT
Mmp12	Forward: TCTGCTGAAAGGAGTCTGCAC<br>Reverse: AGGTTTCTGCTGGGAACCTTCA

Lgals3	Forward: TACTAGAAGCGGCCGAGC<br>Reverse: TGTCTGCCATTTTCCTGGGTA
Lrp1	Forward: CAAAGCTGAAGGCTCCGAGT<br>Reverse: TATGCGGACACTCTCATCGC
Tgfb1	Forward: TGGAGCAACATGTGGAACTC<br>Reverse: GTCAGCAGCCGGTTACCA
Tgfb2	Forward: AGGAGTGGCTTCACCACAAAGACA<br>Reverse: ATTAGACGGCACGAAGGTACAGCA
Tgfb3	Forward: AGCTCTTCCAGATACTTCGACC<br>Reverse: AAAGACAGCCATTCAGCGGT
Timp1	Forward: CATGGAAAGCCTCTGTGGATA<br>Reverse: CTCAGAGTACGCCAGGGAAC
Postn	Forward: AGAGAAATCCCTGCACGACA<br>Reverse: GTTGGTGCAAACAAGGTCCA
Bcl2	Forward: CACCCCTGGTGGACAACATC<br>Reverse: GTTCCACAAAGGCATCCCAGC
Col1a1	Forward: TGACTGGAAGAGCGGAGAGTA<br>Reverse: GAGTAGGGAACACACAGGTCT
Col3a1	Forward: CTGTAACATGGAAACTGGGGAAA<br>Reverse: CCATAGCTGAACTGAAAACCACC
Casp3	Forward: AGCTTGGAACGGTACGCTAA<br>Reverse: GAGTCCACTGACTTGCTCCC
Casp8	Forward: GCGTGGAACAGGAAGTGAGTA<br>Reverse: GAAGAGCTGTAACCTGTGGC
Bok	Forward: CAGCGTATACCGGAACGTGG<br>Reverse: TTGCCCCATGTGATACCTGC
Bak	Forward: CAAGATCGCCTCCAGCCTATT<br>Reverse: CCCAGGAAGCCGGTCAAAC
Bid	Forward: CCGCAAACCTTTGCCTTAGC<br>Reverse: AACCGTTGCTGACCTCAGAGT
Bad	Forward: AAATGGGAACCCCAAAGCAG<br>Reverse: GAACATACTCTGGGCTGCTGG
Puma (Bbc3)	Forward: GAGACAAGAAGAGCAGCATCG<br>Reverse: TAGTTGGGCTCCATTTCTGG
Bax	Forward: ACAGGGGCCTTTTTGCTACA<br>Reverse: CACTCGCTCAGCTTCTTGGT
Noxa (Pmaip1)	Forward: ATAACTGTGGTTCTGGCGCA<br>Reverse: CAATCCTCCGGAGTTGAGCA
Apoa5	Forward: AACCGAGCAGGGGCCAT<br>Reverse: TAGTCCCAGAGGCTCTTCCG
Pnpla2	Forward: AATCTCTACCGCCTCTCGAA<br>Reverse: AGGGTTGGGTTGGTTCAGTA
Angptl3	Forward: TTTGGGAGGCTCGATGGAGAA<br>Reverse: TGCTTGCTGTCTTTCCAGTCT
Angptl4	Forward: GACTGCCAGGAACTCTTCCAA<br>Reverse: GTCCAGCCTCCATCTGAAGT
Lpl	Forward: TACCCCCTAGACAACGTCCA<br>Reverse: CCAGCTGGATCCAAACCAGT
Gpihbp1	Forward: CACAGCGGAACCGACAAAG<br>Reverse: GACTGGCAACAGGTCTGAGT

Agpat1	Forward: AACCTTTGTCGGTTCCGCT<br>Reverse: GACAGAGATACAGCCAGCCG

TaqMan probes

Gene	TaqMan Assay ID
Gapdh	Mm99999915_g1
Vcl	Mm00447745_m1
Myh7	Mm0060555_m1
Nppb	Mm01255770_g1
Nppa	Mm01255748_g1
Acta1	Mm00808218_g1
Acta2	Mm00725412_s1
Atp2a2	Mm00437634_m1
Myh6	Mm00440354_m1
Myh7b	Mm01249977_m1

Online Table 2

Summary of STAR Alignment

Genotypes	Total number of reads	Uniquely mapped reads (N)	Uniquely mapped reads (%)
WT	56,986,661	44,080,962	77.35
WT	58,592,357	45,042,018	76.87
WT	57,741,248	43,997,161	76.2
WT	47,657,324	36,515,508	76.62
WT	51,350,429	36,020,507	70.15
Myh6-Mcm	49,365,176	38,120,087	77.22
Myh6-Mcm	55,698,208	42,618,724	76.52
Myh6-Mcm	52,784,899	37,604,823	71.24
Myh6-Mcm	54,496,135	41,523,760	76.2
Myh6-Mcm	62,083,142	46,430,324	74.79
Myh6-Mcm:*Ctnnb1*^{LoF}	49,258,626	37,285,368	75.69
Myh6-Mcm:Ctnnb1^{LoF}	55,434,509	43,330,362	78.16
Myh6-Mcm:*Ctnnb1*^{LoF}	56,104,421	43,793,695	78.06
Myh6-Mcm:*Ctnnb1*^{LoF}	66,455,498	51,492,977	77.48
Myh6-Mcm:*Ctnnb1*^{LoF}	51,960,626	41,176,883	79.25
Myh6-Mcm:*Ctnnb1*^{GoF}	57,822,245	46,206,202	79.91
Myh6-Mcm:*Ctnnb1*^{GoF}	52,708,415	41,106,319	77.99
Myh6-Mcm:*Ctnnb1*^{GoF}	48,100,763	37,181,898	77.3
Myh6-Mcm:*Ctnnb1*^{GoF}	48,171,139	37,186,377	77.2
Myh6-Mcm:*Ctnnb1*^{GoF}	53,481,970	40,409,473	75.56

Abbreviations: WT: wild type; *Myh6:Mcm*: Myosin heavy chain 6:MerCreMer; Ctnnb1: β-catenin; LoF: Loss-of-function; GoF: Gain-of-function.

Data in the WT and *Myh6-Mcm* groups are from Rouhi et al. [33]
